# Spatial omics in the AI era: Technologies, algorithmic ecosystems, biological applications, and large model perspectives

**DOI:** 10.1002/imt2.70146

**Published:** 2026-07-06

**Authors:** Haoxiu Wang, Xinwang Yang, Siheng Wang, Zhe Yang, Xiuhui Yang, Yutong Yang, Zirong Li, Yuqi Ren, Qianqian Zhang, Bowen Zhao, Jingming Xiao, Yidong Wang, Junhao Dong, Zhenhao Kou, Jie Li, Liqun Yang, Erhu Zhao, Gregory Fonseca, Ruibang Luo, Mingyu Yang, Hongjuan Cui, Gengjie Jia, Dan Wang, Haoyang Li, Jun Ding, Zhiyuan Yuan, Haojing Shao

**Affiliations:** ^1^ State Key Laboratory of Genome and Multi‐omics Technologies, Shenzhen Branch, Guangdong Laboratory of Lingnan Modern Agriculture, Genome Analysis Laboratory of the Ministry of Agriculture and Rural Affairs, Agricultural Genomics Institute at Shenzhen Chinese Academy of Agricultural Sciences Shenzhen China; ^2^ State Key Laboratory of Resource Insects, Medical Research Institute Southwest University Chongqing China; ^3^ MOE Key Laboratory of Computational Neuroscience and Brain‐Inspired Intelligence, MOE Frontiers Center for Brain Science Fudan University Shanghai China; ^4^ Westlake University Hangzhou China; ^5^ Guangdong Provincial/Zhuhai Key Laboratory of IRADS Beijing Normal‐Hong Kong Baptist University Zhuhai China; ^6^ McGill University Montreal Canada; ^7^ City University of Hong Kong Hong Kong China; ^8^ Peking University Beijing China; ^9^ Nanyang Technological University Singapore Singapore; ^10^ Department of Medical Sciences, College of Medicine and Health Sciences Khalifa University Abu Dhabi United Arab Emirates; ^11^ The University of Hong Kong Hong Kong China; ^12^ Yale University New Haven USA; ^13^ Stanford University Stanford USA

**Keywords:** algorithms, biological applications, large models, spatial omics, technologies

## Abstract

Spatial omics technologys help overcome key limitations of conventional omics approaches that lack spatial information, by providing a panoramic perspective from the molecular level to the microenvironment scale for addressing spatially resolved biological questions in life sciences. With the rapid advancement of this field, there are significant differences among technology platforms, algorithms, and research workflows, which bring three core challenges to interdisciplinary researchers: the detailed explanation of technical principles, the selection of appropriate algorithms, and the future development directions. This review systematically summarizes the technical platforms and analytical algorithms of spatial omics, compares their advantages and disadvantages in the context of specific tasks and presents application cases across multiple biological fields. It also outlines the emerging research directions and advances in large model integration. It ultimately aims to provide a reference for researchers from diverse disciplines to design and implement spatial omics studies.

## Highlights

- Systematically reviews recent spatial omics and their core computational tasks.
- Critically assesses algorithm scenarios, bottlenecks, and recommended strategies.
- Comprehensively outlines spatial omics applications in biomedicine, neuroscience, and plant/animal sciences.
- Outlines future trends in 3D reconstruction, virtual cells, temporal modeling, and large language models.

## Graphical abstract

This review focuses on spatial omics, covering the introduction and comparison of technology platforms, explanation and recommendation of algorithm ecology, demonstration of biological applications, and prospect of large models. It aims to help researchers in the interdisciplinary field of spatial omics quickly understand the current situation and research pain points, and establish a comprehensive perspective on spatial omics.

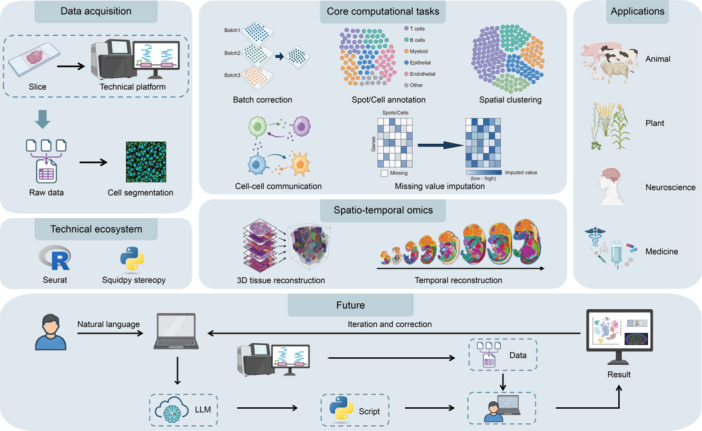

## INTRODUCTION

Humans and many other eukaryotes are composed of billions of highly heterogeneous cells [1, 2]. The intracellular regulation of cell state follows a hierarchical cascade from the genome, epigenome, transcriptome, and proteome to the metabolome, accompanied by bidirectional crosstalk and reciprocal regulation across all molecular layers, which together form the macroscopic regulatory network of life activities [3]. At present, single‐cell technologies targeting single omics have become increasingly mature, and have emerged as the preferred approach for characterizing the status of complex tissues and dissecting mechanisms in life sciences [4, 5, 6, 7, 8, 9, 10, 11, 12, 13, 14, 15]. However, the biological activities of organisms rely on a highly ordered spatial microenvironment. The loss of spatial positional information in conventional single‐cell technologies has severely limited researchers' deep understanding of complex biological systems [16]. Spatial omics have partially compensated for the limitations of conventional omics approaches, such as the loss of spatial location information and the averaging of microenvironmental heterogeneity. Notably, spatial transcriptomics, which for the first time integrally combines single‐cell resolution with spatial location information, was named Method of the Year in 2020 by Nature Methods [17]. Similarly, spatial proteomics has been recognized as Method of the Year in 2024 for its groundbreaking advances enabling systematic analysis of the protein composition and spatial distribution patterns of tissues and organs [18].

Meanwhile, current research on spatial omics continues to thrive in the era of artificial intelligence [19, 20, 21, 22, 23, 24]. A variety of technical platforms, the complexity of analytical algorithms, and the substantial differences in biological applications have greatly increased the obstacles for interdisciplinary researchers to carry out research [25]. Therefore, a series of review articles [3, 26] have elaborated on the technical schemes and specific applications of spatial omics, while multiple benchmarking studies have summarized the advantages and disadvantages of spatial omics analysis software. This review not only provides a general introduction to these technologies but also synthesizes existing review and benchmarking articles, aiming to offer comprehensive recommendations for spatial omics researchers in terms of platform selection, software selection, and future research directions.

**Figure 1:**
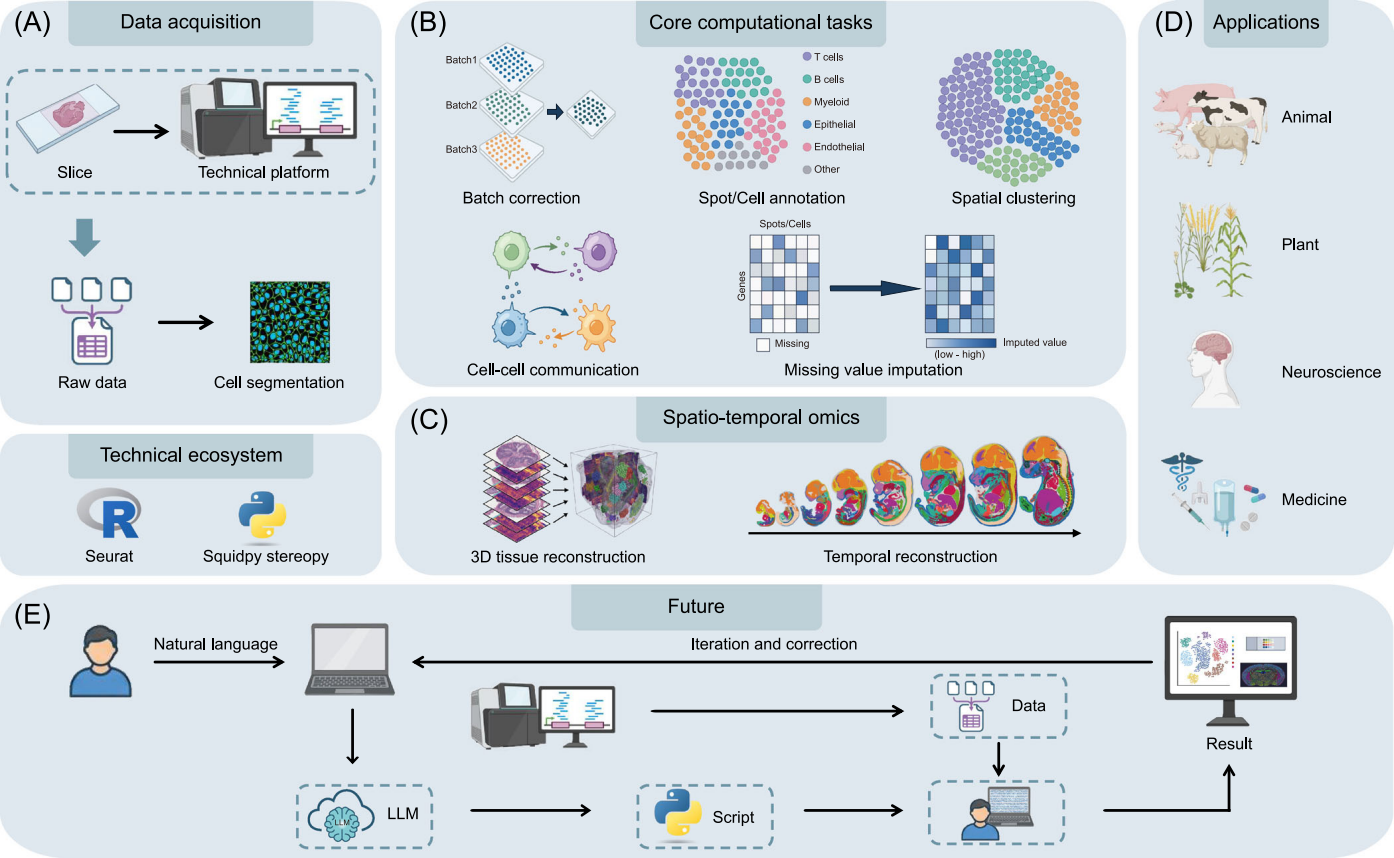
The logical framework of this review follows the data flow of spatial omics research, ranging from data generation and data analysis to scientific discovery. (A) Data acquisition stage of spatial omics. (B) Core computational tasks in the field of spatial omics. (C) Cutting‐edge development direction: Spatio‐temporal omics. (D) Biological applications of spatial omics (medicine, neuroscience, zoology, and botany). (E) It demonstrates that researchers in future spatial omics can leverage LLMs to bypass coding procedures for scientific research, and highlights the tremendous potential of large models in advancing spatial omics research. LLM, Large Language Model.

This review follows the data flow of spatial omics research, from data generation and data analysis to scientific discovery (Figure 1). Firstly, it will systematically introduce the working principles and horizontal comparisons of mainstream technical platforms, as well as their cell segmentation methods (Figure 1A). Then, it will discuss the two common data structure systems of Python and R. Subsequently, from raw data processing to biological interpretation, this review will conduct an in‐depth analysis of core computational workflows, including spatial domain identification, cell annotation, cell communication, batch correction, and imputation (Figure 1B). On this basis, it will further focus on three‐dimensional spatial reconstruction and spatiotemporal dynamic analysis [27, 28, 29] (Figure 1C). Afterwards, breakthrough research achievements of spatial omics in animals, plants, neuroscience, and clinical fields are summarized [30, 31, 32] (Figure 1D). Finally, the opportunities and challenges in the field of spatial omics are elaborated with a focus on the currently prevalent generative models and large models (Figure 1E).

## SPATIAL OMICS TECHNOLOGIES

Spatial omics technical platforms are diverse [27, 28, 29, 30]. Currently, mainstream spatial omics technologies each possess distinct technical characteristics, advantages, and limitations, and are respectively suitable for different research scenarios and detection requirements. Truly simultaneous multi‐omics profiling on the same tissue section remains scarce in current research making absolute direct comparisons difficult. This review systematically summarizes the relevant information concerned by users of several spatial omics technology platforms for comparison (Table 1).

**Table 1:** Performance characteristics demonstration of a comprehensive spatial omics technology platforms.

Technology	Resolution	Core principle	Sample compatibility	Distinctive features
Sequencing‐based spatial transcriptomics				
Visium	55 μm	Slide‐based spatial transcriptomics for *in situ* mRNA capture and whole‐transcriptome quantification	Human, mouse, rat; fresh, frozen, and FFPE tissues	Mature commercial workflow, high data reproducibility
Array‐seq	30 μm	Modified oligonucleotide microarray for spatial transcriptomics with barcoded mRNA capture	Human, mouse; fresh, frozen, and FFPE tissues	Higher resolution, larger area, lower cost, comparable sensitivity vs Visium
DBiT‐seq	10 μm	Orthogonal microfluidic barcoding for spatial co‐mapping of transcriptome and proteome	Human, mouse; fresh, frozen, and FFPE tissues	Multi‐omics integration (transcriptome and proteome)
HDST	2 μm	2 μm barcoded beads for high‐definition spatial transcriptome sequencing	Human and mouse fresh‐frozen tissues	Ultra‐high‐ resolution
Nova‐ST	10 μm	NovaSeq nanopattern‐based HDMI barcoding for high‐res spatial transcriptomics	Mouse fresh‐frozen tissue samples	Enables large tissue section analysis and multi‐chip spatial mapping
Pixel‐seq	1 μm	1 μm polony gel array, V‑seg for single‑cell spatial transcriptomics	Mouse fresh‐frozen tissues	Ultra‐high resolution
Seq‐Scope	0.5–0.7 μm	Repurposed flowcell, two‑step sequencing for sub‑μm spatial transcriptomics	Mouse fresh‐frozen tissues	Ultra‐high resolution
Slide‐seq	10 μm	Barcoded bead array for whole‐transcriptome RNA spatial profiling	Mouse fresh‐frozen tissues	High sensitivity, near single‐cell resolution
SM‐Omics	100 μm	Slide barcoded probe array for transcriptome‐proteome spatial co‐localization	Mouse fresh‐frozen tissues	Spatial co‐localization of transcriptome and proteome
Stereo‐seq	220 nm	Lithographic DNB array for single‐cell and subcellular spatial transcriptomics	Human/mouse fresh, frozen and FFPE tissue sections	Ultra‐high resolution with mature commercial workflow
Visium HD	2 μm	2 μm continuous barcode square array, high‐density spatial barcoding transcriptome profiling	Human/mouse fresh‐frozen, fixed‐frozen, and FFPE tissues	Ultra‐high resolution, mature commercial workflow
Imaging‐based spatial transcriptomics				
Xenium	Subcellular	Dual‐target validation, RCA amplification and fluorescence decoding for high‐res spatial RNA/protein localization	Human, mouse; fresh, frozen, and FFPE tissues	Imaging‐based, FFPE‐compatible, commercialized, Ultra‐high resolution and high‐sensitivity
MERSCOPE	Subcellular	Combinatorial labeling, error‐correcting barcodes and sequential imaging for in situ RNA spatial quantification	Human, mouse; fresh, frozen, and FFPE tissues	Imaging‐based, FFPE‐compatible, commercialized, Ultra‐high resolution and high‐sensitivity
CosMx SMI	Subcellular	Probe/antibody targeting, cyclic imaging‐decoding for high‐throughput spatial RNA/protein quantification	Human, mouse; fresh, frozen, and FFPE tissues	Imaging‐based, FFPE‐compatible, commercialized, Ultra‐high resolution and high‐sensitivity
MERFISH	Single‐cell	Binary barcode probes, sequential imaging for high‐throughput RNA spatial quantification	Human samples	Overcomes limited genes and severe error accumulation in traditional smFISH
seqFISH+	Imaging‐based	Pseudo‐color barcodes and diluted mRNA for RNA quantification via confocal microscopy	Human and mouse fresh and frozen samples	Pseudo‐color channels aided sequencing, ultra‐high spatial resolution
BARseq	Single‐cell	Barcode padlock probes, RCA and in situ sequencing for single‐cell gene mapping and cell typing	Fresh mouse samples	High‐resolution, low detection efficiency and long sequencing cycle
FISSEQ	Subcellular	Preserves morphology, RNA‐to‐cDNA amplicons, in situ sequencing for subcellular gene expression mapping	Human, mouse, rat, Drosophila, and other species	High‐resolution, low detection efficiency and long sequencing cycle
Spatial proteomics				
Spatial‐CITE‐seq	25 μm	ADTs (Antibody‐Derived Tags) combined with microfluidics for 25 μm resolution protein‐transcriptome co‐localization	Human/mouse fresh and frozen tissue sections	ADTs for protein‐specific sequencing, dual microfluidic loading for spatial info
GeoMx DSP	No fixed resolution	WTA probes for human/mouse protein‐coding genes, UV‐cut AOIs tags for sequencing quantification	Human, mouse; fresh, frozen, and FFPE tissues	Bind target mRNA/protein, UV‐cut index tags for sequencing, flexible AOIs
3D CyCIF	Imaging‐based, 3D resolution: 140 × 140 × 280 nm	30–50 µm thick tissue sections, iterative staining‑imaging‑quenching cycles, confocal 3D reconstruction for high‑resolution 3D protein imaging	Human/mouse and FFPE samples	High‐resolution 3D structure
MIBI‐TOF	Imaging‐based	Metal isotope‐labeled antibodies + mass spectrometry for subcellular protein‐element spatial analysis	Human formalin‐fixed paraffin‐embedded (FFPE) samples	Metal isotope‐labeled antibodies, distinct from conventional fluorescent antibodies
DESI‐MSI	—	Optimize DESI parameters, combine FAIMS and MS for protein imaging and identification	Fresh tissue, human and mouse	Charged droplet probe combined with MS for protein analysis and identification
MALDI‐MSI	—	MALDI imaging mass spectrometry integrates histology for spatial correlation of molecules and morphology	FFPE, frozen and fresh tissues from human, mouse, rat, bovine, etc.	Label‐free, simultaneous analysis of multiple proteins; wide species applicability
Spatial metabolomics				
LAESI‐MSI	—	Atmospheric‐pressure IR laser ablation ionization enables metabolite qualitative‐quantitative and spatial imaging	Fresh plant samples	Mass spectrometry‐based Spatial Metabolomics
Spatial epigenomics				
Spatial‐ATAC‐seq	20 μm	Spatial chromatin accessibility sequencing at cellular level via in situ Tn5 and microfluidic barcoding	Fresh, frozen tissue sections, human and mouse samples	Similar principle to Spatial‐CITE‐seq; spatial information via microfluidics
Spatial‐CUT&Tag	20 μm	Spatial histone modification profiling via in situ CUT&Tag, microfluidic barcoding and sequencing	Mainly human, mouse fresh and frozen tissue sections	Similar to Spatial‐CITE‐seq; spatial information via microfluidics

Abbreviations: ADT, antibody‐derived tag; AOIs, areas of interest; DNB, DNA nanoball; FFPE, formalin‐fixed paraffin‐embedded; RCA, rolling circle amplification.

### Spatial transcriptomics technology

Spatial transcriptomics technologies [30, 31, 32, 33] can be classified into sequencing‐based and imaging‐based categories according to their principles. Sequencing‐based spatial transcriptomics takes next‐generation sequencing (NGS) [34, 35] as its core strategy, and achieves in situ capture and localization of molecules via spatial barcoding. Imaging‐based spatial transcriptome technology directly detects RNA molecules in tissue sections by fluorescence in situ hybridization (FISH) [36] or in situ sequencing (ISS) [37], without physical isolation or sequencing steps.

### Sequencing‐based spatial transcriptomics technologies

Most sequencing‐based spatial transcriptomics platforms use in situ capture arrays coupled with high‐throughput sequencing. These methods encompass various technical principles and application scenarios but share a basic spatially probe/barcode based system. Stereo‐seq [38, 39] achieves large field‐of‐view and even subcellular‐resolution transcriptomic profiling by employing ultra‐high‐density, spatially barcoded DNA nanosphere arrays [40]. Seq‐Scope utilizes high‐resolution spatial barcode arrays built on Illumina flow cells, achieving submicron resolution of 0.5–0.7 μm through a dual‐sequencing workflow [41]. HDST [42] applies a split‐pool approach to generate 2 μm silica beads arranged in a hexagonal array, attaining subcellular resolution of 2 μm via multiple rounds of fluorescence decoding, providing a powerful tool for high‐resolution analysis of healthy and diseased tissues. Slide‐seq and Slide‐seqV2, based on barcoded bead arrays, have been instrumental in reconstructing developmental trajectories of the mouse hippocampus and cortex at 10 μm resolution [43]. Pixel‐seq, leveraging polony gel technology [44], resolves cellular heterogeneity and region‐specific molecular changes at 1 μm resolution, making it suitable for mapping complex tissues. Nova‐ST exploits the nanopatterned surface of Illumina NovaSeq flow cells and is widely compatible with diverse species and tissue types due to its low cost, high resolution, and high capture efficiency [45]. Visium is commonly used in clinical and basic research, offering standardized protocols compatible with both fresh‐frozen and FFPE samples. Unlike the full transcriptome coverage strategies described above, GeoMx DSP supports targeted transcriptome analysis of regions of interest based on probe‐based optical cut tags. microSTRS [46] combines enzymatic in situ polyadenylation, enabling simultaneous processing of bacterial and host RNAs. MAGIC‐seq [47] employs an innovative grid microfluidic chip combined with carbodiimide chemistry and a novel spatial barcode, facilitating 3D spatial transcriptomics (ST) analysis.

### Imaging‐based spatial transcriptomics technologies

Imaging‐based spatial transcriptomics technologies mainly fall into two categories: in situ sequencing and in situ hybridization with both using microscope detected fluorescence mapping.

Among in situ sequencing approaches, BARseq [48] is a high‐throughput method built on Illumina sequencing‐by‐synthesis chemistry. It simultaneously targets endogenous mRNAs and virally delivered synthetic RNA barcodes in tissue sections, amplifies signals via rolling circle amplification, and performs in situ sequencing with spatial coordinate recording. Fluorescent in situ sequencing (FISSEQ) [49] generates cDNA through in situ reverse transcription, immobilizes cDNA via chemical cross‐linking to prevent diffusion, and forms three‐dimensional amplicon matrices by rolling circle amplification. In situ sequencing is then performed via ligation‐based sequencing under a confocal microscope. Xenium is an in situ imaging platform based on in situ hybridization (ISH), padlock probe ligation, and rolling circle amplification (RCA) for signal enhancement.

Within the in situ hybridization category, multiplexed error‐robust fluorescence in situ hybridization (MERFISH) [50] uses combinatorial labeling and optimized error‐robust Hamming coding schemes, coupled with sequential hybridization and imaging. osmFISH is a non‐barcoded, amplification‐free cyclic single‐molecule FISH technique that achieves linear expansion of targeted transcripts through repeated hybridization and wash cycles. seqFISH+ [51] alleviates optical crowding via pseudocolor encoding, combined with multiple rounds of barcoded hybridization including error correction, as well as hydrogel embedding and tissue clearing. Ultrasensitive Sequential Fluorescence In Situ Hybridization (USeqFISH) [52] integrates hydrogel‐based tissue clearing, dual signal amplification via rolling circle amplification and hybridization chain reaction, and toehold‐mediated sequential probe stripping. CosMx [53] is a spatial multi‐omics platform that uses cyclic ISH and oligonucleotide‐conjugated antibodies for direct fluorescence detection.

### Comparison of commercial spatial transcriptomics technologies

Several reviews have compared the advantages and disadvantages of commercial ST technologies [25, 54, 55, 56]. Generally, the seven most common commercial platforms include Slide‐seqV2, Stereo‐seq, Visium V1, Visium HD, Xenium, CosMx, and MERSCOPE (Table 2). Sequencing‐based ST platforms are primarily designed for discovery purposes, offering versatility applicable to all species but facing challenges such as diffusion and limited capture capacity. In contrast, image‐based ST platforms focus on precise insights, with core advantages including low false discovery rates, high gene expression detection, and accurate cell segmentation; however, their gene capture range is narrow and often limited to human and mouse samples.

**Table 2:** Horizontal comparison of commercial spatial omics platforms.

Commercial technology	Resolution	Number of genes profiled	Chip Size	Species	Advantages	Disadvantages	Cell segmentation
Slide‐seqV2	10 μm	Whole transcriptome	3 mm diameter	Any species	High sensitivity	Diffusion effect, small capture area	Normal
Stereo‐seq	0.22 μm	Whole transcriptome	10 mm × 10 mm	Any species	Large chip, high‐ resolution, capable of simultaneous microbial detection	Diffusion effect, requires fresh‐frozen tissue	Normal
Visium HD	2 μm	Whole transcriptome	11 mm × 11 mm	Human and mouse only	High detection sensitivity and specificity	Diffusion effect, relatively low sequencing saturation	Normal
Visium V1	55 μm	Whole transcriptome	6.5 mm × 6.5 mm	Any species	Mature protocol, widely applied	Diffusion effect, low resolution	NA
Xenium	Subcellular	5001	10.45 mm × 22.45 mm	Human and mouse only	Extremely low FDR, stable performance on archived FFPE tissues, excellent reproducibility	Relatively high operational barrier, relatively high cost	Best
CosMx	Subcellular	6175	20 mm × 15 mm	Human and mouse only	High number of detected genes, low on‐board operational barrier	Relatively high FDR	Best
MERSCOPE	Subcellular	1000	18 mm × 22 mm	Any species	Supports whole‐tissue scanning	Low gene panel, relatively high FDR	Good

*Note*: Focusing on the horizontal comparison of commercial spatial omics technology platforms, the evaluation indicators cover resolution, gene throughput, chip size, applicable species, advantages, limitations, and cell segmentation performance.

### Cell segmentation in spatial transcriptomics

Spatial omics technologies have overcome the spatial information bottleneck of traditional single‐cell sequencing. However, raw data from these platforms only contain discrete molecular coordinates and lack clear cell boundary information. Thus, cell segmentation [57, 58, 59] is a critical bridge connecting molecular detection and single‐cell analysis [60]. ST platforms such as Stereo‐seq, Xenium, and CosMx all provide cell segmentation as part of their delivery results. Several studies have evaluated these built‐in cell segmentation outcomes and found that Xenium and CosMx perform exceptionally well [25, 54, 55]. At the raw data level, images from image‐based ST are multimodal, including staining of cell nuclei, cell membranes, and cytoplasm. Notably, cell membrane or cell wall staining is the gold standard for detecting cell boundaries: the F1 score for cell boundary detection using cell membrane or cell wall staining is generally above 0.9 [61], whereas methods such as nuclear extension typically score below 0.8 [62]. Therefore, multimodal cell segmentation incorporating cell membrane or cell wall staining can significantly improve accuracy.

Some platforms such as Visium HD do not have default cell segmentation software, and users can also choose software independently to improve accuracy. Cell segmentation faces challenges such as blurred boundaries in dense tissues, misjudgment of low‐convexity cells (including neurons, macrophages, and some migrating cells), poor cross‐modal adaptability, and high computational costs.

Four core concepts are commonly involved in ST cell segmentation: nucleus segmentation, transcript assignment, whole‐cell segmentation, and full cell‐boundary reconstruction. Nucleus segmentation involves identifying and outlining the cell nucleus, typically using a DNA stain such as 4′,6‐diamidino‐2‐phenylindole staining. Transcript assignment is a computational step in spatial transcriptomics where individual RNA molecules are linked to specific cells. This approach is generally applied to technologies with nuclear staining but no explicit boundary staining, requiring algorithms that extend from cell nuclei, such as SCS [63] and Proseg [64]. Whole‐cell segmentation defines the entire outer boundary of a cell and requires markers for the plasma membrane or cell wall to include areas beyond the nucleus. Full cell‐boundary reconstruction involves 3D modeling of the physical limits, extending beyond 2D outlines to account for cell volume, irregular shapes, and overlapping layers in thick tissue samples. Since users typically focus on assigning all transcripts to individual cells in single slices, this section collectively considers both transcript assignment and whole‐cell segmentation as cell segmentation.

Existing tools are mainly divided into image‐based and transcriptome‐integrated categories, among which Cellpose [65], Baysor, SCS, and Proseg have their own characteristics. Cellpose is a universal deep learning‐based segmentation tool that converts segmentation into a gradient vector field prediction task, adopts an optimized U‐Net [66] structure, and supports cross‐modal and 3D segmentation. Its disadvantage is that it performs generally on low‐convexity cells, and the Cellpose3 [67] version further improves the processing capability of noisy and blurred images. Proseg is an unsupervised probabilistic model inspired by the Cellular Potts Model, which requires initialization with nuclear staining, can correct transcript localization, and performs excellently in segmenting tumor‐infiltrating immune cells with higher computational efficiency than similar tools. However, it relies on nuclear staining and lacks GPU acceleration.

Baysor realizes image‐transcriptome joint segmentation. Based on Markov Random Fields (MRF) and Bayesian Mixture Models (BMMs), it can rely solely on transcriptome data or combine auxiliary staining signals, with few parameters and easy operation, adapting to various ST platforms and dense tissues. SCS adopts a deep learning architecture, uses the watershed algorithm to identify cell nuclei as initial references, aggregates high‐dimensional gene expression information from neighboring regions through a Transformer network [68, 69], and forms cell boundaries by combining gradient flow tracking [70], enabling high‐precision segmentation and excelling in fine characterization of cell boundaries. The four tools are suitable for different scenarios and can be flexibly selected according to the availability of nuclear staining, the need for integrating transcriptome information, available computational resources, and segmentation accuracy requirements.

### Spatial proteomics technologies

ST technologies primarily provide indirect measurements of cellular states, as most biological processes are controlled by proteins. The emergence of spatial proteomics technology provides researchers with the possibility of directly analyzing cellular life phenomena [71]. Current spatial proteomic technologies [72, 73, 74] have developed into a diversified system, consisting of three types: spatial transcriptome‐proteome co‐analysis, highly multiplexed protein imaging, and mass spectrometry‐based in situ imaging.

In spatial transcriptome‐proteome co‐analysis, Spatial‐CITE‐seq employs antibody‐derived DNA tags and spatial barcodes to achieve simultaneous spatial profiling of the whole transcriptome and highly multiplexed proteins at 25 μm resolution, suitable for fresh‐frozen tissues but not for FFPE samples [75]. GeoMx DSP is based on probe light cutting labeling technology, which can accurately quantify transcripts and proteins in space [76]. This platform exhibits broad sample compatibility, supporting FFPE specimens, fresh frozen tissues, as well as normal, tumor, lesional, and embryonic tissues. However, it shows relatively low compatibility with fibrotic, lipid‐rich, and calcified tissues.

Highly multiplexed protein imaging technologies enable in‐depth dissection of tissue microenvironments at single‐cell or subcellular‐resolution. CODEX [77, 78, 79, 80] supports theoretically unlimited multiplexing via DNA‐barcoded antibodies and cyclic imaging. 3D CyCIF [81] achieves three‐dimensional localization of dozens of proteins using thick tissue sections and cyclic immunofluorescence, reducing artifacts inherent to 2D imaging. Immuno‐SABER [82] provides programmable signal amplification and super‐resolution imaging compatible with diverse samples. MIBI‐TOF [83] uses metal isotope‐labeled antibodies to achieve high‐sensitivity, subcellular‐resolution spatial analysis of proteins and endogenous elements, particularly in FFPE tumor and neural tissues.

Mass spectrometry‐based in situ imaging supports label‐free spatial molecular analysis. DESI‐FAIMS‐MS [84] enables efficient desorption and spatial localization of intact proteins in fresh‐frozen tissues. MALDI‐MSI [85] performs label‐free imaging of peptides, small molecules, and proteins up to 25 kDa (up to 110 kDa in optimized protocols) and is compatible with FFPE and frozen samples. PLATO [86] integrates artificial intelligence deep learning algorithms with microfluidic technology to achieve high‐resolution spatial proteome profiling at the whole‐tissue section level (25 μm resolution, thousands of proteins), breaking through the bottleneck of high‐throughput in situ omics technologies.

### Spatial metabolomics and spatial epigenomics technologies

Beyond spatial transcriptomics and proteomics, spatial epigenomics and metabolomics [87] can directly and functionally reflect cellular physiological processes and behaviors, including core molecular activities such as glycolysis, DNA methylation, and histone modifications. LAESI‑MSI [88], Spatial‑ATAC‑seq [89], and Spatial‑CUT&Tag [90] are key technologies in spatial metabolomics and spatial epigenomics. LAESI‑MSI can perform in situ qualitative, quantitative, and spatial distribution analysis of metabolites in fresh, intact plant tissues and hydrated biological samples at atmospheric pressure, without the need for matrix coating or complex sample preparation, and can also achieve in situ metabolic profiling at the single‐cell level [91]. Both Spatial‑ATAC‑seq [89] and Spatial‑CUT&Tag are spatial epigenomic technologies. The former relies on in situ Tn5 transposition and microfluidic dual barcoding to realize spatially resolved analysis of genome‑wide chromatin accessibility in fresh frozen tissue sections, yielding spatially defined single‑cell epigenomic data. The latter, based on in situ CUT&Tag combined with two‑dimensional spatial barcoding, allows genome‑wide spatial analysis of histone modifications at 20 μm single‐cell resolution. It is not restricted by species, applicable to frozen sections of various animal tissues, and supports expanded imaging areas and spatial multi‐omics integration. Together, these three technologies provide robust support for in situ, multi‐dimensional, and high‐resolution spatial multi‐omics research from the perspectives of spatial metabolic phenotypes, chromatin accessibility, and histone modifications.

### Summary and outlook

Currently, spatial omics technologies are rapidly emerging, demonstrating great promise in the life sciences [92]. Although some technologies have been commercialized, most are still in the laboratory stage and cannot be used on a large scale. Moreover, most current technologies are still limited to single‐omics detection, which greatly restricts multi‐omics joint analysis. In the future, multi‐omics simultaneous detection methods such as DBiT‐seq [93] and DBiTplus [94], may become commercially available, enabling researchers to comprehensively understand cells at multi‐omics and multi‐dimensional levels without relying on multi‐modal integration [95, 96]. However, such technologies face inherent technical barriers—including incompatible sample preparation requirements across omics layers, destructive iterative processing, and intrinsic mismatches in resolution and signal readout. Collectively, these challenges hinder the implementation of in situ multi‐omics analysis on single tissue sections, representing a critical bottleneck that urgently requires resolution. In summary, spatial omics technologies have established an increasingly mature framework and delivered remarkable scientific insights, yet significant room for advancement and critical technical challenges remain to be urgently addressed.

## THE COMPUTATIONAL ECOSYSTEM OF SPATIAL OMICS

**Figure 2:**
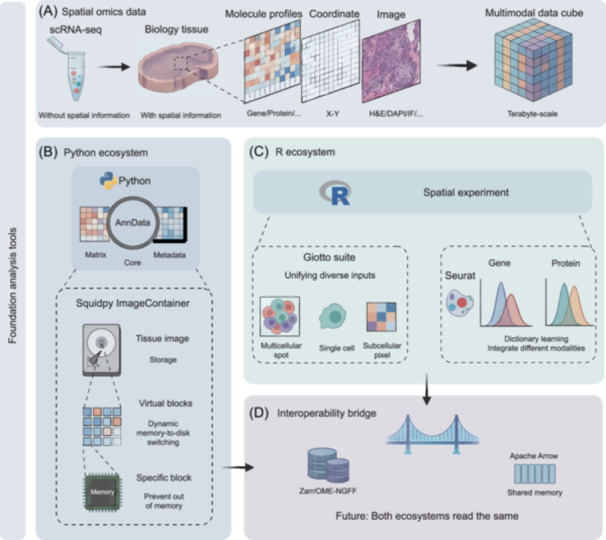
The computational ecosystem and analytical workflow of spatial omics. (A) Spatial omics data. (B) The Python computational ecosystem centers on the AnnData structure, which tightly encapsulates the molecular expression matrix with biological metadata (left). This foundation extends to Squidpy (First published in 2022), Squidpy's ImageContainer resolves by utilizing dynamic memory‐to‐disk switching to load massive high‐resolution tissue images as manageable virtual blocks into RAM (right). (C) The R computational ecosystem is anchored by the SpatialExperiment class. Within this ecosystem, the Giotto (first published in 2013) suite unifies diverse spatial inputs while Seurat integrate multiple modalities (first published in 2013). (D) Cross‐language interoperability between Python and R ecosystems relies on universal standard formats such as Zarr, OME‐NGFF, and Apache Arrow.

The introduction of spatial dimensions brings specific computational challenges that stem from the multimodal structure of spatial omics data (Figure 2A). Traditional single‐cell analysis relies only on molecular abundance matrices [97]. In contrast, spatial analysis fundamentally requires integrating these molecular profiles with precise physical coordinates. Furthermore, many spatial workflows integrate high‐resolution histological images, such as hematoxylin‐eosin stained or immunofluorescence labeled images. The combination of these diverse data types significantly increases the overall data volume and computational requirements [98]. Analyzing a single tissue slice using high‐resolution platforms like Stereo‐seq generates terabytes of raw data, rendering traditional single‐cell software paradigms inadequate [99, 100, 101].

Spatial omics platforms now achieve single‐cell or subcellular‐resolution, generating millions of data points per tissue section. Mapping these high‐density molecular signals onto physical coordinates produces high‐dimensional data matrices that exceed the capacity of basic visual overlays. To systematically extract complex biological patterns, the field requires computational infrastructures capable of efficiently storing, manipulating, and integrating these multimodal data layers. Consequently, researchers have established two primary software ecosystems based on Python and R [102]. These parallel infrastructures provide standardized data structures and foundational frameworks, driving the transition from descriptive spatial mapping to quantitative spatial omics analysis.

The Python ecosystem provides standardized infrastructures for spatial omics analysis. Figure 2B illustrates this ecosystem. The cornerstone of this ecosystem is Scanpy [103]. Originally developed for single‐cell analysis, Scanpy provides highly efficient algorithms for essential tasks, including dimensionality reduction, cell clustering, and graph‐based operations. Scanpy achieves its high performance through a specialized data structure called AnnData [104]. AnnData organizes complex biological information into a single, cohesive object. It stores the main molecular expression matrix alongside detailed metadata for individual cells and specific genes. By keeping the data matrix and its annotations tightly linked, AnnData prevents data fragmentation during complex analyses. Because of its structural efficiency, AnnData has become the universal foundation for almost all Python‐based spatial omics tools [105].

Scanpy stores physical coordinates for two‐dimensional visualization but processes them as standard numerical features [106]. Squidpy [107] extends the Scanpy framework to compute spatial topological relationships and integrate high‐resolution imaging data. First, Squidpy converts spatial coordinates into spatial neighborhood graphs by determining physical adjacency between cells or spots. This topological structure enables specific spatial statistical analyses to quantify the physical organization and interaction of distinct cell types within the tissue. Second, Squidpy solves the massive memory bottleneck caused by high‐resolution histology images. Platforms such as Visium HD and Xenium generate tissue images that can reach gigabyte or even terabyte sizes. Traditional data structures attempt to load an entire file into the active memory (RAM) at once, which exceeds the capacity of standard hardware. Squidpy introduces a novel data architecture called the ImageContainer. It divides large tissue images into smaller spatial blocks, loading specific blocks from disk to RAM only during algorithmic execution. This block‐wise processing integrates spatial graphs with gigapixel images under strict memory limits.

The Python ecosystem also provides essential preprocessing tools for imaging‐based spatial transcriptomics. Imaging technologies generate multi‐channel fluorescent images. To translate these raw optical signals into quantitative molecular counts, developers created the Starfish framework. This framework operates directly on this raw visual data to extract quantitative molecular counts. First, it aligns multiple tissue images through image registration. Next, it removes technical noise via background subtraction. Finally, it applies decoding algorithms to identify specific molecules. By mathematically resolving overlapping fluorescent spots, Starfish can determine the precise physical location of each transcript. These consecutive operations transform optical pixels into a standard gene expression matrix. This matrix serves as the input for downstream spatial modeling modules.

Parallel to Python, the R ecosystem provides standardized infrastructures for spatial omics analysis (Figure 2C). Deeply anchored in the Bioconductor project, the R ecosystem provides distinct advantages in rigorous statistical testing and multi‐scale data integration. This computational landscape is predominantly shaped by three foundational pillars: the Giotto Suite, Seurat v5, and the SpatialExperiment class [108]. Giotto [109] processes data across more than 50 spatial modalities, including imaging mass cytometry, spatial proteomics, and spatial transcriptomics. Different spatial platforms generate multi‐cellular spots, isolated single cells, or sub‐cellular pixel blocks. Giotto standardizes these inputs by defining specific spatial boundaries for each data point. It converts spots, cells, and pixels into a unified coordinate framework. This framework enables continuous multi‐scale analyses from broad tissue regions to sub‐cellular details. Seurat serves as another major foundation for R‐based spatial analysis. It specializes in integrating multiple biological modalities. Seurat executes multimodal data integration to link spatial coordinates with single‐cell reference atlases. Different data types, such as mRNA counts and protein fluorescence intensities, exhibit distinct statistical distributions. Seurat aligns these modalities using dictionary learning algorithms. These algorithms construct a shared mathematical space to capture non‐linear correlations between distinct modalities. This alignment projects single‐cell reference data onto spatial tissue spots to map proteins or genes in physical locations. The SpatialExperiment class functions as the standard data container within the R ecosystem. It extends the SingleCellExperiment framework by introducing dedicated storage compartments for physical spatial coordinates and histology images. This standardized structure allows different R‐based statistical tools to read and process identical datasets without object conversion [110, 111, 112].

Spatial omics workflows frequently integrate tools across Python and R ecosystems. This cross‐language integration requires data structure conversion. Algorithms must translate AnnData objects into Seurat or SpatialExperiment objects. This object conversion duplicates high‐dimensional data matrices within active memory. Furthermore, structural disparities between these language‐specific containers cause metadata loss and coordinate misalignment during data transfer. These computational constraints currently restrict seamless cross‐language interoperability.

Tools such as the SpatialData framework aim to standardize object transformations and represent currently practical solutions for cross‐language workflows. However, migrating data between different languages remains a significant technical challenge. To truly address this, researchers should move beyond reliance on cumbersome, language‐specific wrappers. Instead, the field needs a common, foundational standard that can run on all programming platforms. However, abandoning wrappers entirely is impractical due to fundamental differences in object models between Python and R. Developers should build lightweight Application Programming Interfaces that seamlessly package common data formats into user‐friendly native structures, such as AnnData in Python or SpatialExperiment in R. We propose two critical directions to drive this computational unification moving forward (Figure 2D). The first direction involves changing how data is stored and shared. Currently, software tools attempt to load massive datasets entirely into the active memory. To mitigate this, future proposals suggest adopting modern on‐disk storage formats like Zarr or OME‐NGFF (Figure 2D). Conceptually, these formats divide massive data into smaller blocks and store them directly on the hard drive. Furthermore, for in‐memory operations, adopting language‐neutral formats like Apache Arrow has been proposed. In theory, Arrow would allow Python and R to share the exact same memory buffer without time‐consuming serialization, extracting only the specific data blocks needed. However, it is crucial to note that these unified formats (Zarr/OME‐NGFF/Arrow) are primarily speculative future trends. In current routine workflows, they have not yet matured enough to provide seamless, out‐of‐the‐box cross‐language interoperability for end‐users. To make this conceptual unification a practical reality, developers must first build optimized lightweight tools in both languages that can read these shared files directly and smoothly. The second direction is standardizing how physical locations are recorded. Spatial biology urgently requires a universal protocol to manage coordinates across different scales. Python and R often handle spatial locations differently. Developers from the Python scverse ecosystem and the R Bioconductor foundation should collaborate to solve this. They need to define a strict and shared set of rules for storing physical coordinates. Establishing this universal standard ensures that spatial alignments and cell boundaries remain mathematically identical across platforms. This guarantees consistent results, whether a researcher uses a deep neural network in Python or a statistical model in R.

With robust data structures and cross‐language platforms established, the computational focus shifts toward deep biological inference. Researchers employ a flexible modeling repertoire tailored to the specific scales and modalities of the tissue data. For instance, graph‐based networks are frequently adopted to discretize cellular neighborhoods and establish spatial topologies. In parallel, statistical frameworks such as Gaussian process regression excel at modeling continuous spatial gradients and identifying spatially variable genes. When high‐resolution histology is available, deep learning architectures can be integrated to extract latent morphological features.

The strength of these diverse approaches lies in their ability to capture spatial dependencies. They achieve this through various mathematical tools, such as spatial kernels that model distance‐decaying relationships or graph convolutions that aggregate local neighbor information. By optionally aligning these spatial datasets with single‐cell transcriptomic reference atlases, researchers can transcend the resolution limits of individual capture spots. Ultimately, these problem‐driven computational modules allow for a customized exploration of complex tissue mechanisms. These customized workflows range from cell type deconvolution and spatial domain identification to distance‐constrained cell–cell communication (CCC). By leveraging these adaptable tools, researchers move beyond descriptive snapshots toward predictive spatial biology.

## KEY COMPUTATIONAL TASKS IN THE FIELD OF SPATIAL OMICS

**Figure 3:**
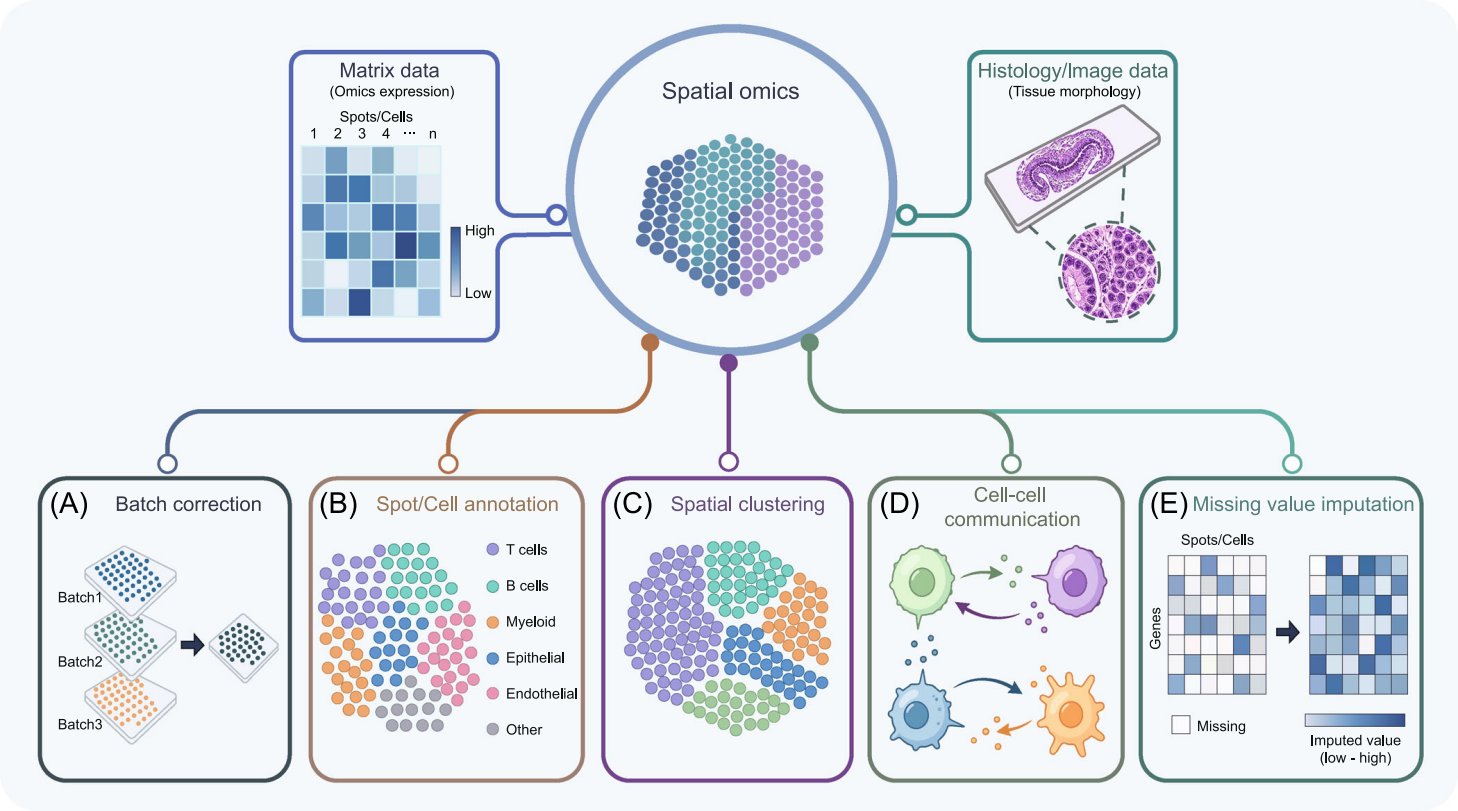
Key computational tasks in the field of spatial omics. (A) Batch correction. (B) Cell annotation (spatial deconvolution). (C) Clustering analysis (spatial region identification). (D) Cell–cell interaction analysis. (E) Spatial omics data imputation.

The key computational tasks in the field of spatial omics mainly include: spatial clustering, cell type annotation, intercellular spatial communication analysis, batch effect correction, and missing value imputation (Figure 3). This section will systematically organize and evaluate relevant software from different computing tasks, providing researchers with suitable research plans.

### Spatial clustering

#### Introduction

Cluster analysis serves as a fundamental cornerstone of spatial omics analysis. It primarily partitions cells and spots into distinct clusters based on feature similarity, enabling spatial region identification, cell type annotation, and the dissection of tissue heterogeneity. Current studies predominantly employ the Leiden and Louvain algorithms for cell clustering. However, these conventional clustering methods rely solely on gene expression profiles while neglecting spatial location and morphological features. Though adequate for basic single‐cell annotation tasks, they tend to produce biologically implausible clustering outcomes in the spatial domain and tissue microenvironment analyses, such as grouping spatially distant spots and cells into the same cluster. In contrast, spatial clustering algorithms that integrate spatial information and histological image features can effectively address this limitation. Accordingly, this section systematically summarizes the technical characteristics of prevailing spatial clustering approaches, conducts comprehensive performance evaluations using large‐scale benchmark datasets, and provides targeted practical guidelines for their implementation in spatial omics research.

#### Classification of clustering methods in spatial omics

According to algorithm design principles, current spatial omics clustering methods can be divided into three categories: Bayesian statistical clustering, graph neural network clustering, and deep learning fusion clustering.

Bayesian statistical methods incorporate spatial proximity as prior information into the clustering process by constructing probability models, making them suitable for fine structure analysis of high‐resolution data. BASS [113] adopts a Bayesian hierarchical modeling framework to achieve synchronous clustering of cell types at the single‐cell scale and spatial domain detection at the tissue region scale. This method supports multi‐slice and multi‐scale analysis, demonstrating excellent performance in transcriptome profiling analysis of complex tissues such as the cerebral cortex and hypothalamus, especially suitable for single‐cell resolution spatial omics data. Its core advantage lies in the ability to reveal cell states that depend on the microenvironment and maintain stable clustering accuracy in highly sparse data. BayesSpace [114] is based on a full Bayesian statistical model, which utilizes spatial neighborhood information to enhance clustering resolution and identify organizational structures that cannot be detected at the original resolution. This method has been validated in various tissue samples such as brain tissue and melanoma, and is particularly adept at analyzing transcriptional heterogeneity. However, this method is computationally time‐consuming.

Graph neural network methods effectively capture spatial dependencies and nonlinear interactions by constructing spatial neighborhood maps of cells/spots, aggregating local information through graph convolution and other operations. SpaGCN [115] integrates gene expression, spatial location, and histological information to identify spatial domains by generating undirected weighted maps. This method aggregates the expression features of neighboring spots through graph convolution, which can detect more differentially expressed genes with spatial enrichment patterns. It has fast computation speed and does not rely on any platform, making it suitable for various ST datasets. GraphST [116] is a graph neural network method based on supervised contrastive learning, which learns discriminative features by minimizing the embedding distance of spatially adjacent spots. This method performs outstandingly in the fine structural depiction of brain tissue and embryonic tissue, and supports multi‐slice joint analysis and batch effect correction, making it one of the preferred methods for Visium data. STAGATE [117] adopts a graph attention autoencoder framework to adaptively learn the similarity of adjacent spots through the attention mechanism, effectively characterizing the features of spatial domain boundaries. This method preserves spatial expression patterns while denoising data, supports three‐dimensional spatial domain extraction with multiple continuous slices, and has excellent computational efficiency, making it an ideal choice for large‐scale data processing.

Deep learning fusion methods integrate domain adversarial networks and other technologies to achieve multimodal data fusion and complex feature extraction, suitable for diverse spatial omics data types. DeepST [118] first extracts morphological image features through pre‐trained deep neural networks, and integrates gene expression and spatial position data to construct a spatial enhanced expression matrix. Then, the graph neural network (GNN) autoencoder and denoising autoencoder are combined to generate spatially enhanced latent representations, and DAN is used to achieve multi batch/cross technical data integration. At the same time, this method supports 3D spatial domain extraction and batch effect correction, and is particularly adept at identifying heterogeneous subregions within visually homogeneous regions of tumor tissue, demonstrating the strongest stability in biological replicates and spatially perturbed samples. BANKSY [119] unifies cell type clustering and tissue domain recognition tasks by embedding cells into the product space of their own transcriptome and local neighboring transcriptome. The highlight of this method is its support for RNA and protein imaging data, which is fast and scalable, capable of processing datasets of millions of cells, and can be used for quality control and spatially aware batch effect correction. SpaceFlow [120] generates spatially consistent low‐dimensional embeddings based on spatially regularized depth map networks, and integrates pseudo temporal and spatial positional information through pseudo spatiotemporal (PST) maps. This method is highly effective in trajectory inference and spatiotemporal pattern analysis, supporting divide and treat strategies for processing large‐scale data and adapting to research scenarios such as cardiac development and tumor immune interactions. CCST [121] is based on unsupervised graph convolutional network methods, focusing on de novo cell clustering and subtype discovery, which can clearly identify cell cycle stages and functional cell subtypes. This method performs the best in intestinal tissue and imaging data, with strong spatial domain continuity, and is the first choice for research that emphasizes marker gene mining. Spatial‐MGCN [122] reconstructs the expression matrix based on multi view graph convolutional network method and zero dilation negative binomial decoder, and performs well in clustering and trajectory inference tasks of high‐resolution data (such as MERFISH/Xenium). CellCharter [123] focuses on an algorithm framework for identifying and characterizing cellular microenvironments, capable of analyzing the microenvironment features of millions of cells across data types and technologies. The SEDR [124] embedding method, which combines deep autoencoders and graph autoencoders, performs the best in maintaining spatial continuity and is suitable for scenarios with high sparse data and high requirements for spatial consistency. This method has strong resistance to disturbances caused by neighborhood merging or adding clusters, but its adaptability to complex spatial forms is slightly inferior to DeepST and BASS.

#### Analysis of clustering benchmark test results

Benchmark testing of spatial omics clustering methods has now established a mature evaluation framework. Among representative studies, Chen et al. [125] constructed a large‐scale benchmark comprising 600 real and simulated datasets across 10 technical platforms and 8 organ types, conducting systematic multidimensional validation of 14 mainstream methods. Yuan et al. [126] focused on method complementarity and scenario adaptation, clarifying optimal selection strategies for different data types and task objectives. The evaluation metrics in these studies primarily address three core dimensions: clustering accuracy, spatial continuity, and computational efficiency. Key application scenarios include spatial domain recognition, cell type clustering, and spatial niche detection. The findings are organized into five perspectives to guide researchers in selecting appropriate clustering tools.

Significant differences in data characteristics across technology platforms directly influence the adaptability of clustering methods. The Visium platform is the most commonly used sequencing technology, with STAGATE and GraphST performing best, followed closely by BASS and BayesSpace. On the Slide‐seq platform, STAGATE, SpaGCN, and BASS represent top‐tier methods. For high‐resolution imaging platforms such as MERFISH, Xenium, CosMx, BASS, BANKSY, and Spatial‐MGCN deliver leading performance. For sparse data like seqFISH+, PRECAST performs relatively well, although its overall accuracy remains modest.

These two studies also found that tissue spatial complexity significantly impacts clustering method performance. Different organ types require targeted tool selection: for layered organs such as brain tissue, STAGATE and GraphST achieve the highest accuracy and clearly depict cortical layering. Methods adapted for intestinal tissue, including CCST, DeepST, and CellCharter, effectively analyze spatial heterogeneity in intestinal villi. In breast and heart tissues, stLearn, PRECAST, and BayesSpace perform better. PRECAST and stLearn handle dispersed spatial distributions in low‐continuity organs such as liver and lungs more effectively. For research requiring high spatial consistency, SEDR, SpaceFlow, and BASS are optimal choices.

In practical research, the influence of biological repetition and spatial disturbance cannot be ignored, and the stability of the method needs to be carefully considered. For biological repetition samples, DeepST, BANKSY, SEDR, GraphST, and STAGATE have the best accuracy and stability. DeepST, SEDR, STAGATE, and GraphST exhibit the strongest resistance to perturbation scenarios caused by neighborhood merging or adding clusters. It is worth noting that the method using strong spatial smoothing assumption is more sensitive to boundary offset, and the performance drops significantly in perturbed samples. Therefore, caution should be exercised when selecting analysis software and setting parameters.

Data characteristics are the key factors governing method selection. Their influence follows a descending order: data sparsity, number of clusters, and spatial morphology. Data sparsity acts as the most critical determinant; as sparsity increases, the accuracy of most methods decreases substantially. Among them, BASS, BANKSY, and DeepST are the most robust to high sparsity data, while PRECAST, SpaGCN, and STAGATE are relatively sensitive. As the number of clusters increases, the accuracy of all methods decreases linearly. In terms of spatial form adaptability, DeepST, BASS, and SEDR can handle all forms such as linear, circular, and mosaic, while some methods such as SpaGCN have limited performance in irregular forms.

The computational efficiency directly affects the applicability of the method in large‐scale data, and there are significant differences between different types of methods: Bayesian methods usually take longer time and occupy more memory due to the iterative calculation requirements of probability models. GNN/deep learning methods achieve fast inference and more stable efficiency through network optimization. From the perspective of influencing factors, the number of data points mainly affects the running time, while sparsity mainly affects the computational accuracy rather than efficiency. GNN/deep learning methods can be prioritized for large‐scale data processing.

#### Tool recommendation and summary

Researchers can select scenario‐based methods by combining different data types, organ/tissue types, and research objectives. STAGATE, GraphST, BASS, DeepST, and SEDR are the top five methods in terms of comprehensive performance, covering most mainstream application scenarios (Table 3). Their core adaptation directions can accurately match research needs: STAGATE is suitable for Visium data, large‐scale data processing, and 3D spatial domain extraction, especially for scenarios that pursue computational efficiency. GraphST performs outstandingly in layered organ (such as brain) analysis, fine structure depiction, and multi‐slice batch calibration. BASS is a universal and versatile method that is suitable for various scenarios such as high‐resolution technology, multi‐slice joint analysis, and highly sparse data. DeepST has significant advantages in biological duplicate sample analysis, spatial perturbation resistance, and tumor heterogeneity subregion identification. SEDR is more suitable for research scenarios with high sparse data and high requirements for spatial continuity.

**Table 3:** Software recommendations for key computational tasks.

Task	Software	Language	Advantages
Clustering	BASS [113]	R	Simultaneous clustering and spatial domain detection, adapts to high‐sparsity data, reveals microenvironment‐dependent states, strong versatility
	STAGATE [117]	Python	Adaptive neighborhood similarity, balances denoising and spatial patterns, efficient computation, compatible with 10× Visium and large‐scale data
	GraphST [116]	Python	Superior boundary detection in brain/embryo tissues (top‐ranked on 10× Visium). Unique batch effect correction. No predefined genes needed
Cell annotation/deconvolution	cell2location [127]	Python	Integrates multi‐batch/cross‐platform data, accurately resolves cell composition in mixed spots
	RCTD [128]	R	Multi‐modes for high/low‐resolution data, strong versatility
	Tangram [129]	Python	Single‐cell resolution reconstruction, compatible with multiple technologies, low memory, high speed
Batch correction	Harmony [130]	R	Strong versatility, assists STAGATE/SEDR for batch calibration
	STAligner [131]	Python	Same/cross‐platform data integration, high alignment accuracy
	SpaCross [132]	Python	Efficient multi‐slice integration and batch correction
Cell communication	Cellchat [133]	R	Fast speed, low memory, supports quantitative signal/pathway analysis, stable results
	stLearn [134]	Python	Morphology‐standardized expression data, identifies ligand‐receptor interaction hotspots, excellent performance in brain/tumor data
Data Interpolation	gimVI [135]	Python	Optimal imputation for moderately sparse Visium data, accurately fills technical missing values
	MAGIC [136]	Python	Simple efficient algorithm, adapts to high‐density sparse data (e.g., Stereo‐seq), low computational cost

Although significant progress has been made in spatial clustering methods, there are still three core challenges that need to be addressed based on benchmarked test results. Firstly, current methods are not effective at identifying small and disconnected spatial domains. In complex tissues such as tumors and liver tissue, these methods often fail to accurately detect small groups of cells that are scattered across different regions. Secondly, there is a lack of ability in multi‐slice joint analysis and 3D reconstruction. Most methods focus on single slice analysis, making it difficult to effectively integrate spatial information from multiple consecutive slices. The third bottleneck is the efficiency of large‐scale data processing. For datasets with millions of cells, some methods have problems with high memory usage and long running time. Overall, the selection of spatial omics clustering methods requires comprehensive consideration of various factors such as data types, organizational characteristics, research objectives, and computational resources. There is no universal optimal method, and researchers need to use methods in conjunction with practical scenarios.

### Cell annotation/deconvolution

#### Introduction

Annotation is a necessary path from omics data to biological interpretation. If the technical resolution reaches single‐cell resolution, researchers can directly classify clusters based on marker genes or map them using reference datasets (SingleR [137], Garnett [138]). However, currently many spatial omics techniques can only achieve Spot level resolution, so it is crucial to use deconvolution methods that break down a mixed spot into proportions of different cell types to complete annotation tasks. At present, there are many deconvolution algorithms, making it difficult for researchers to quickly choose the most suitable tool for personal research. Therefore, this section will describe the principles and evaluation of deconvolution software to assist researchers in their selection.

#### Deconvolution tool

Based on whether they rely on single‐cell RNA sequencing (scRNA‐seq) reference data, ST deconvolution methods can be divided into three categories: reference dependent, reference independent, and ensemble methods. Each method, with its unique algorithm design and technical advantages, is adapted to the ST data analysis needs in different scenarios.

The deconvolution method relying on scRNA‐seq reference is based on existing single‐cell data, and uses multiple models to achieve cell type analysis and feature mining of spatial spots. cell2Location [127] uses a Bayesian hierarchical model to integrate multi‐source data, with the core advantage of being able to integrate multiple batches and cross‐platform data. RCTD [128] provides multi‐resolution adaptation modes. Doublet mode (assign 1−2 cell types to each sequencing point) adapts to high‐resolution technologies such as Slide‐seq and MERFISH. Full mode (assign any number of cell types to each sequencing point) adapts to low‐resolution technologies such as Visium. Multi‐mode supports multi cell‐ type resolution. DestVI [139] is based on a conditional deep generative model, which can not only restore the proportion of cell types, but also capture cell type specific snapshots of the transcription status at each cell, supporting downstream differential expression analysis and molecular feature extraction. STRIDE [140] supports multi‐dimensional downstream analysis based on late Dirichlet allocation modeling. In contrast, Stereoscope [141] does not require data normalization or gene selection. It directly estimates negative binomial distribution parameters based on scRNA‐seq data and can reuse single‐cell parameters to analyze multiple spatial datasets without repeated training. SpatialDecon [142] optimizes the deconvolution effect through background correction and specific gene screening. In the non‐negative matrix factorization class, SPOTlight [143] initializes Non‐negative Matrix Factorization regression with cell type labeled genes, combined with non‐negative least squares deconvolution, which is suitable for low‐quality data. SpatialDWLS [144] performs outstandingly in accuracy and running speed based on weighted least squares optimization. DSTG [145] of graph model class utilizes graph convolutional networks to fuse transcriptional similarity and spatial relationships, and SD^2^ [146] innovatively integrates dropout features to improve the deconvolution accuracy of low‐resolution data. The deep learning framework Tangram [129] can align sc/snRNA‐seq data with various spatial data (MERFISH, STARmap, Visium, histological images, etc.). The core advantage lies in the ability to achieve single‐cell resolution spatial gene expression map reconstruction, correct low‐quality genes, provide single‐cell level resolution for low‐resolution technologies, and support spatial mapping of multimodal data (such as SHARE seq). The optimal transmission class SpaOTsc [147] and NovoSpaRc [148] efficiently complete the correlation analysis between Single cells and spatial positions through mapping construction and probability allocation, respectively. In addition, CARD [149] combines non negative matrix factorization and conditional autoregressive models to explain the spatial correlation of cell‐ type composition, and also supports deconvolution analysis without reference data.

Other methods do not require prior acquisition of single‐cell data, and instead directly mine cell type features from the ST data. Among them, STdeconvolve [142] is based on an unsupervised LDA model, which performs better than partially reference dependent methods without reference data and is suitable for various space technology platforms. SpiceMix [150] uses probabilistic latent variable modeling to combine spatial information with gene expression to reveal cell identity and differentiation trajectories. Berglund et al. [151] also developed a novel algorithm that can extract tissue component specific expression profiles without the need for single‐cell data, which can accurately distinguish between healthy and diseased areas and is suitable for spatial analysis of tumor tissues.

The ensemble method, represented by EnDecon [152], adopts a weighted ensemble learning strategy to integrate multiple basic deconvolution results. The assigned weights are highly positively correlated with the performance of the basic method, which not only demonstrates better performance than a single method in simulation studies, but also effectively identifies spatial co localization patterns of multiple cell types, significantly enhancing the stability and accuracy of the deconvolution results.

#### Tool evaluation

The tool principle is complex, but researchers can choose appropriate methods based on experimental details such as whether single‐cell data is used, whether multimodal data is available, resolution size, and so forth. However, the cost of software replacement is still high, so the reference section of this section recommends tools for researchers based on existing benchmark tests [153, 154]. Based on accuracy testing, researchers recommend using cell2Location, RCTD, and spatial DWLS. In robustness testing, CARD, cell2Location, Tangram, and SD^2^ have strong tolerance to changes in gene number, resolution, cell type number, normalization method, and hyperparameters. From the perspective of computational efficiency, NMFreg, STRIDE, and Tangram are the fastest. Tangram also has the advantage of low memory usage. Stereoscope runs the slowest and consumes the highest amount of memory. For rare cell types, a simple marker gene signature scoring approach often outperforms more complex models [155]. From the perspective of users, CARD, cell2Location, RCTD, and DestVI have the most detailed documentation, strong code reusability, and low difficulty in getting started. Based on multiple benchmark tests, CARD, cell2Location, and Tangram have shown leading performance in accuracy, robustness, and usability, and are suitable for most research scenarios, making them the first choice for researchers.

Different ST techniques and research needs can be matched to choose more advantageous analysis software: low‐resolution techniques (such as Visium) recommend using RCTD adapted to Full mode, as well as SPOTlight with marker gene initialization and stable accuracy. High‐resolution technologies such as Slide‐seq and MERFISH can use Doublet mode or Tangram that supports single‐cell resolution reconstruction. If pursuing result stability, the ensemble method EnDecon can be chosen, which can integrate the advantages of the top 3 single methods. When conducting 3D tissue reconstruction, priority should be given to using STRIDE, which supports native continuous slice integration and 3D reconstruction. Tangram can adapt to multi‐modal data space mapping such as SHARE seq to meet the integration requirements of RNA chromatin accessibility and other multimodal data. For intercellular signal transduction analysis, it is recommended to use SpaOTsc, which can infer the spatial distance of signal transduction and intercellular communication.

### Cell communication tools

#### Introduction

Cell communication is the core process in multicellular organisms where cells exchange information and co‐regulate life activities through ligand receptor recognition and signal transduction [156, 157]. Spatial genomics has spawned a diverse array of specialized tools for CCC analysis, each tailored to distinct data characteristics, analytical goals, and technical requirements. These tools integrate spatial information with ligand‐receptor interaction data to varying degrees, and their core strengths range from complex characterization and functional consequence prediction to quantitative signaling analysis and high‐resolution data adaptation.

CellPhoneDB [158] is a classic tool dedicated to intercellular communication mediated by ligand‐receptor complexes. Its core advantage lies in considering the subunit structures of ligands and receptors, accurately representing heterologous complexes, and combining statistical frameworks to predict interactions enriched between cell types from single‐cell transcriptome data. The early v2.0 version [159] enabled functions including supplementation of interaction molecules and optimized query efficiency for large datasets, and it required 2 h to process a dataset of around 10 GB. Previously, researchers had to import Scanpy‐processed h5ad data into the R environment and run CellPhoneDB based on Seurat objects. This workflow was cumbersome and easily caused errors during repeated data format conversions. The latest v5 version [160] has thoroughly solved the above problems. It natively supports Python and achieves seamless compatibility with Scanpy AnnData objects. In addition, it adds more than 200 experimentally verified interactions, greatly enriching information on immune checkpoint and cytokine‐related interactions. For researchers without programming experience, the built‐in CellPhoneDB Viz interactive web visualization module enables zero‐code operation, supporting result interaction viewing, conditional filtering, and chart export, so as to intuitively display ligand–receptor‐based CCC analysis results.

#### Tool overview

stLearn [134], NicheNet [161], and CytoTalk [162] represent a suite of specialized tools developed with distinct methodological designs and application focuses. stLearn integrates three types of data: spatial, morphological, and gene expression. It standardizes gene expression data through morphological similarity and neighborhood smoothing, reconstructs the spatial transformation gradient of cell types using PST distance, and can also identify tissue hotspots with high ligand receptor interaction activity. It is suitable for analyzing cell communication in combination with tissue morphology and demonstrates high accuracy in brain slice and tumor dataset analysis. As one widely adopted method, NicheNet stands out for its unique strength in predicting the functional outcomes of CCC, instead of merely annotating ligand‐receptor pairs. It combines the expression data of interacting cells with prior knowledge of signal transduction and gene regulatory networks to infer the impact of active ligands on target cell gene regulation, and is suitable for exploring the role of communication in pathways or phenotypes. CytoTalk takes integrated intracellular and intercellular gene networks as inputs and constructs cell type specific signaling networks from scratch using reward‐based Steiner Forest algorithm. After verification with ST data, it performs well in signal pathway construction and cross tissue and cross developmental stage comparative analysis.

COMMOT [163], CellChat [133, 164], and ICELLNET [165] have emerged as powerful solutions for quantitative assessment and spatially aware modeling of CCC. COMMOT is based on the collective optimal transport model, considering the competition between ligands and receptors and the spatial distance between cells. It can output spatial signal vector fields, gradients, and other results, clearly visualizing the source and destination of signals. It is suitable for research involving signal gradients such as development and tumor invasion. CellChat is based on the CellChatDB ligand receptor database, using the law of mass action to calculate communication probabilities. Combined with permutation testing, a weighted directed communication network is constructed, which can achieve quantitative signal analysis and pathway level resolution, with fast running speed and low memory usage. ICELLNET integrates a ligand receptor database compiled by experts, supports communication score quantification, and can associate target cell populations with 31 reference human cell types, providing three visualization modes that have been experimentally validated to effectively analyze cell communication regulation mechanisms.

CellNEST [166] and spaCI [167] are optimized for high‐resolution data and complex scenes. CellNEST introduces a relay network communication detection method that can identify potential communication patterns of ligand receptor ligand receptor, detect signal pathways in specific tissues, and provide interactive visualization functions, suitable for analyzing complex communication at the single‐cell level. The effectiveness of the tool has been validated in tumor samples and relevant researchers can refer to it for use. SpaCI is an adaptive graph model that combines attention mechanisms to integrate cell spatial location and gene expression profiles. It can effectively handle data loss and noise issues, identify active L‐R signaling pathways and upstream transcription factors between adjacent cells, and perform well in sparse data and tumor sample analysis. This method has been validated for its superior performance in seqFISH+ data of mouse cortex and NanoString CosMx spatial molecular imaging (SMI) data of non‐small cell lung cancer samples.

#### Tool evaluation and recommended workflow

An evaluation based on the Spatial Transcriptome Distance Enrichment Score (DES) [168] demonstrates that CCC methods constructed on statistical models exhibit the best overall performance, with CellChat, ICELLNET, CellPhoneDB, and NicheNet leading in overall rankings. CCC inference via ligand–receptor (L‐R) analysis based on spatial transcriptomics (ST) faces fundamental limitations. Because RNA abundance does not reliably reflect protein availability or downstream signaling activity, such approaches often produce substantial erroneous predictions [169, 170]. Thus, the consistency of results between different tools is extremely low, and researchers recommend combining at least two tools to achieve highly reliable interactions. Based on this, the following are recommended for different scenarios: CellChat+CellPhoneDB is the preferred choice for general use, suitable for tumor microenvironment (TME) and routine data screening, with stability, speed, and low memory consumption. For studies emphasizing spatial consistency, the recommended priority order follows CellChat, ICELLNET, and NicheNet. When highly credible interactions are required, the combination of CellPhoneDB and ICELLNET is suggested. NicheNet presents clear advantages in exploring the functional consequences of cellular signaling and can be prioritized for such purposes. Large‐scale datasets are more suitably analyzed using CellChat and CellPhoneDB. High‐resolution spatial data can be interpreted with the combined use of CellChat and stLearn. NicheNet, CytoTalk, and stLearn should be avoided for rapid analysis of large datasets. Fast analysis of big data should avoid NicheNet, CytoTalk, and stLearn, while pursuing spatial consistency should avoid Giotto and iTALK [171].

A standardized entry‐level workflow is proposed for novice researchers. Investigators may first conduct preliminary cell type screening using CellPhoneDB. Signal direction and gradient distribution can then be visualized using COMMOT. High‐resolution data supports fine‐grained interaction analysis at the single‐cell level with CellNEST or spaCI. Pathological sections can be deeply characterized using stLearn by integrating morphological information. Advanced users may select suitable tool combinations according to the specific strengths of each software package and their available programming language environments.

### Batch correction

Batch effects frequently influence spatial omics analysis, arising from factors such as sample variability, technical discrepancies, differences in measurement environments, or operational errors. These batch‐related artifacts can obscure biological signals, diminish statistical power, and even lead to misleading or biased conclusions—complicating data interpretation and integration. Therefore, addressing batch effects is critical for reliable data integration and accurate biological inference, particularly in large‐scale, multi‐platform, or multi‐sample studies [172]. Despite the increasing abundance of ST analysis tools, the definition of batch effects remains vague, and there is a lack of systematic evaluation of batch effect correction in ST alignment and integration processes in this field. A study [173] proposed that batch effects can be divided into four categories: inter‐slice variation, inter‐sample variation, cross‐platform bias, and intra‐slice variation. Researchers should consider which type of batch effect the research scenario belongs to, in order to facilitate targeted batch correction.

Currently, methods for comparison and integration in spatial transcriptomics can be categorized into three groups based on their batch effect correction capabilities and underlying algorithms. Several software tools have incorporated batch effect considerations into their initial design, including SLAT, STAligner, GraphST, SpatiAlign, DeepST, and SPIRAL. Some tools partially mitigate batch effects but still require external software (e.g., Harmony [130]) for comprehensive calibration, such as STAGATE, SEDR [124]. In contrast, other software relies entirely on external tools for batch correction, including BASS, BayesSpace, and SpaGCN.

Researchers have tested various spatial omics analysis tools on multiple datasets with distinct batch effect definitions, employing four widely recognized evaluation metrics [174]: graph connectivity (GC), k‐nearest neighbor batch effect test (kBET), integrated local inverse Simpson index (iLISI), and batch average contour width (ASW). DeepST, STAligner [131], GraphST, STitch3D, PRECAST, spatiAlign, and SPIRAL methods were systematically evaluated. The results indicate that no universally optimal method exists. For single‐platform studies, GraphST, PRECAST, STAligner [131], and SPIRAL are recommended. For cross‐platform analyses, SPIRAL and STAligner are preferred. Notably, SpatiAlign exhibits weak performance across most scenarios.

Recent advances have led to the development of specialized batch correction software. For example, SpaCross [132] which achieves efficient Multi‐slice integration based on cross mask graph autoencoder, STAIG [175] which integrates gene expression, spatial coordinates, and histological images using graph contrastive learning without pre alignment to achieve batch correction, and SpaBatch [176] which combines variational graph autoencoder (VGAE), self‐supervised learning, and triplet learning based on readout aggregation strategy to achieve correction. These emerging methods currently lack evaluation experiments, but researchers can use UMAP visualization to remove batch effects and prevent such deep learning methods from causing distortion of biological structures and affecting subsequent analysis.

In summary, batch correction represents a major technical challenge in experimental research. However, if batch effects are excessively strong, conclusions derived from corrected data may still be unreliable. Therefore, it would be advisable to first delineate the specific research questions and define the relevant batch effect type during the batch correction stage [172]. Since most evaluations do not allow direct comparison of absolute correction efficacy, UMAP visualization serves as a valuable tool for verifying effectiveness: uniform mixing of multiple batches indicates a favorable correction outcome. Simultaneously, the intensity of correction should be minimized to avoid distorting inherent biological patterns.

### Missing data interpolation

#### Research background and core requirements

Although sequencing based spatial transcriptome technologies such as Slide‐seq V2 and Stereo‐seq have achieved spatial localization of gene expression, they generally face the problem of extremely low capture rates‐a large number of true expression signals are erroneously recorded as zero values (i.e., loss phenomenon), which seriously interferes with downstream clustering analysis and tissue region identification, and other key research [25]. As the core means of solving this problem, data imputation needs to achieve two key goals simultaneously: accurately filling missing technical values while preserving the true biological zero values to avoid compromising the biological authenticity of the data.

#### Classification and technical characteristics of mainstream interpolation algorithms

The current interpolation algorithms can be divided into two categories based on the data types of core dependencies: cross‐modal interpolation based on single‐cell data and self‐modal interpolation based on spatial information.

Cross‐modal methods utilize paired scRNA‐seq data as reference modalities and achieve missing value completion through cross‐modal alignment. Representative methods include: SpaGE [177] combines Principal Component Analysis (PCA) dimensionality reduction with K‐Nearest Neighbors (KNN) algorithm. This is simple in principle and easy to implement. SpaIM [178] innovatively adopts the style transfer concept, regarding scRNA‐seq data as content and spatial transcriptomics data as style, which effectively eliminates platform discrepancies. StPlus [179] integrates the stDiff module based on the conditional diffusion model with the DISCO module that integrates the GNN dual diffusion mechanism. This method excels in completing large contiguous missing areas. StImpute [180] uses an autoencoder to create gene expression embeddings for ST and scRNA‐seq data. These are used to identify the nearest neighbor cells between scRNA‐seq and ST datasets to infer missing ST data. stDiff [181] uses a conditional diffusion model to predict the missing parts of ST by incorporating the original ST data into the denoising process through two Markov processes.

The self‐modal method does not rely on external reference data, and its core is to construct spatial spots into graph structures, encode spatial information through graph neural networks, and propagate expression signals. Typical representatives include MIST which adopts a two‐step strategy of “spatial region segmentation + intra region expression interpolation”, balancing clustering and interpolation requirements. StAI [182] integrates GNN and autoencoder to achieve “one‐stop” analysis of interpolation and cell type annotation. NovoSpaRc uses the concept of optimal transmission to predict by utilizing the continuity of adjacent cell expression.

#### Evaluation and recommendation

Recent systematic comparative experiments [183] have revealed several key patterns: firstly, there is no universal optimal method, and data type determines adaptability. gimVI [135] performs the best in Visium data (moderate sparsity), Tangram performs the best in Slide‐seq V2 data, while high‐density and sparse data such as Stereo‐seq/sci‐Space are more suitable for MAGIC [136]. Secondly, gene selection strategies significantly impact imputation stability. Using all genes yields better results for high‐density data, whereas selecting 2000–5000 highly variable genes leads to more stable imputation for high‐density sparse data. Thirdly, over‐imputation is a prevalent issue. Except for traditional KNN, most mainstream methods fill all zero values, resulting in near‐zero data sparsity that distorts the true biological sparsity. Finally, computational costs vary drastically and hardware resource constraints cannot be overlooked. Tangram has the highest memory requirements, and gimVI requires the longest running time.

The interpolation of spatial omics data needs to follow a standardized process to ensure a balance between effectiveness and efficiency. Firstly, perform quality control filtering to remove spots and genes with high deletion rates. For mildly sparse data (with low missing rates), the stLearn spatial smoothing method or low rank completion algorithm can be preferred. These methods have low computational costs and can effectively preserve spatial continuity.

However, when paired scRNA‐seq data is available, it is recommended to use cross‐modal interpolation methods such as gimVI, Tangram, or SpaIM. This will achieve the highest accuracy of missing value completion with single‐cell reference information, which is suitable for research scenarios that require high accuracy of results. If there is no paired scRNA‐seq data but the spatial features are significant, it is recommended to use graph neural network‐based methods such as MIST or stAI. These methods can maintain good interpolation performance even without reference data by fully mining spatial proximity information.

When facing data with extremely high missing rates or large blank areas, diffusion models such as stPlus or stDiff should be selected. Their dual diffusion mechanism or conditional diffusion framework are good at handling continuous missing areas, and the completion effect is more coherent. In addition, if quantitative interpolation uncertainty is required for research, TransImpute method can be used, which can output the confidence level of interpolation results for subsequent result verification and reliability evaluation. This will provide uncertainty reference for downstream analysis.

#### Summary

This section provides a systematic summary of software analysis commonly conducted by researchers in multiple spatial omics fields, in order to facilitate users and developers to quickly understand the current status of segmented fields. Table 3 summarizes recommended software for key computational tasks, along with their corresponding programming languages and primary advantages. It can be observed from multiple tasks that no software can achieve comprehensive excellence for a particular task. Therefore, it is recommended that researchers should first consider appropriate tools based on current biological problems when conducting analysis, in order to avoid data losing its biological significance and being unable to conduct effective downstream analysis.

## FROM 2D SLICES TO 3D TISSUE ARCHITECTURE: RECONSTRUCTION AND VIRTUAL CELL MODELING

### Introduction

High‐resolution spatial omics can map the complex molecular structures of tissue microenvironments. Nevertheless, the computational field is currently undergoing a critical methodological transition. Analytical frameworks must evolve from static descriptive mapping to dynamic predictive modeling. Most spatial datasets exist only as two‐dimensional planar snapshots, which can effectively record molecular distributions at a single physical point in time. However, they have two fundamental limitations. First, isolated planar slices are insufficient to capture continuous three‐dimensional anatomical structures. Second, purely observational data cannot mathematically predict dynamic cellular state transitions in response to specific perturbations. To transcend these observational constraints and unlock the predictive capacity of systems biology, the computational ecosystem is rapidly advancing along two parallel and equally critical analytical frontiers: the structural reconstruction of 3D tissue architecture, and the functional simulation of Virtual Cells [184, 185, 186, 187, 188]. These parallel frameworks collectively transform fragmented observational data into dynamic computational models [189], enabling spatial omics research to shift from static spatial description to proactive functional prediction.

### 3D tissue reconstruction

**Figure 4:**
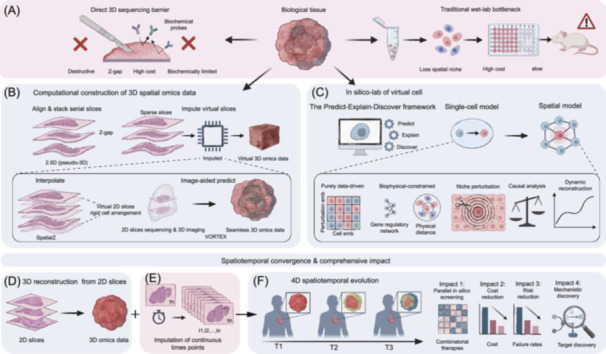
3D spatial omics reconstruction and virtual cell modeling. (A) The intrinsic barriers of direct 3D physical sequencing (left) and the operational limitations of traditional drug and genetic perturbation experiments (right). (B) Computational 3D spatial omics reconstruction methodologies include fragmented 2D slice stacking with Z‐gaps (top left) and complete 3D data imputation (top right), achieved through either mathematical interpolation of virtual slices (bottom left) or morphology‐driven predictive modeling (bottom right). (C) The predict‐explain‐discover (P‐E‐D) framework for single‐cell and spatial perturbations (top). Computational paradigms evolving from purely data‐driven embeddings to biophysically‐constrained models, niche propagation, causal disentanglement, and dynamic spatiotemporal reconstruction (bottom). (D−F) The synergistic integration of 3D spatial reconstruction from discrete 2D slices (D) and continuous temporal imputation (E) constructs patient‐specific 4D virtual organs (F). This unified framework empowers massively parallel in silico screening of combinatorial therapies, uncovering novel molecular mechanisms while drastically reducing exorbitant experimental costs and clinical trial failure rates.

Biological functions inherently operate as continuous three‐dimensional processes. Macroscopic tissue structures, such as neuronal axonal projections and tumor infiltration gradients, span thousands of micrometers [190]. Isolated planar slices fragment this spatial perspective. Researchers must capture complete three‐dimensional architectures to decode these complex tissue heterogeneities. To capture these three‐dimensional structures, experimental technicians have attempted to directly conduct three‐dimensional sequencing studies, using advanced hardware platforms to process thick tissue blocks and directly determine the spatial distribution of molecules at a physical level [191] (Figure 4A). Emerging optics‐free technologies utilize molecular diffusion to encode spatial coordinates directly. However, these direct physical approaches face strict physical constraints. Biochemical reagents struggle to penetrate thick tissue samples. Furthermore, exhaustive serial sectioning irreversibly destroys the entire clinical specimen. Finally, these direct physical measurements generate massive data volumes and high financial costs. To overcome strict physical hardware limitations, researchers have attempted to synthesize three‐dimensional data using mathematical reconstruction methods such as interpolation, aiming to bridge the *Z*‐axis gap in the physical structure of tissue slices. Additionally, some advanced methods combine non‐destructive three‐dimensional morphological imaging with sparse transcriptome sampling to predict complete three‐dimensional expression maps (Figure 4B). While these methods seek to mathematically infer three‐dimensional expression maps without damaging biological samples, they currently represent proof‐of‐concept demonstrations rather than robust biological reconstructions [192].

Parallel to structural reconstruction, the field requires functional prediction of cellular responses to specific perturbations. Previously, analyzing cellular responses to external stimuli primarily relied on laboratory physical experiments. However, these experiments are limited by stringent operating conditions, resulting in long development cycles, significant manpower investment, and high economic costs. Comprehensive studies of thousands of genes are virtually impossible at the experimental level. To overcome these experimental and cost bottlenecks, researchers have constructed computational virtual cell models. These computer simulation systems follow the Predict‐Explain‐Discover (P‐E‐D) framework and can serve as biological flight simulators, conducting large‐scale parallel experiments in a computational environment to mathematically predict cellular phenotypic responses to unknown perturbations (Figure 4C).

Common 3D computation methods rely on contiguous slicing and alignment strategies. Researchers first cut a large tissue into contiguous two‐dimensional slices, and then sort each two‐dimensional slice separately. Finally, they use algorithms to map these contiguous two‐dimensional slices into a unified three‐dimensional coordinate system, thereby achieving 3D reconstruction of the whole tissue. For example, the STAIR framework provides a computational solution for this structural alignment. This method uses a heterogeneous graph attention network to evaluate the gene expression similarity between different two‐dimensional slices. It calculates relative physical distances along the *Z*‐axis based on these similarities. This computational method extracts global structural consistency from existing two‐dimensional datasets and bypasses the need for manual physical distance measurements [193] (Figure 4D).

However, experimental tissue sections themselves have problems with non‐rigid geometric deformation and tissue physical damage, and aligning dozens of sections continuously will lead to a large accumulation of mathematical errors. To address these computational challenges, the Spateo framework has built a computational framework. It uses a generative Gaussian process to explicitly simulate nonlinear tissue deformation and handle tissue loss areas. At the same time, to avoid excessive accumulation of errors, Spateo [194] uses advanced multi‐slice refinement technology, which can jointly align multiple continuous slices. For the *Z*‐axis offset problem, Spateo uses an external morphological 3D mesh as a rigid structural reference and performs discrete optimization, mathematically forcing the stacked spatial slices to accurately match the real external boundary. This deep mathematical integration reduces the artificial spatial displacement along the *Z*‐axis and aims to recover the true anatomical curvature of the original biological specimen.

Structural alignment methods produce a fragmented 2.5D stack of discrete 2D slices. To address the gap problem along the *Z*‐axis, researchers computationally generate missing biological data using mathematical interpolation (Figure 4E,F). This approach synthesizes virtual tissue data to fill in physically empty areas. The SpatialZ framework [195] executes this process. First, SpatialZ calculates the precise physical coordinates of virtual cells based on the cell distribution of adjacent real slices. Next, the framework assigns specific cell types to these new virtual locations. Finally, the algorithm synthesizes a complete gene expression profile for each virtual cell. This virtual generation method bridges physical gaps to construct a continuous and dense 3D cell atlas. This dense structural model eliminates the physical limitations of the original experimental cutting angles, allowing researchers to perform computational cutting at arbitrary angles.

However, this particular mathematical interpolation method suffers from a critical structural flaw at the microscopic level. The algorithm mathematically forces virtual cells to precisely align with the real cells above and below, resulting in an unnatural linear structure where cells are vertically aligned along the *Z*‐axis. Real biological cells do not align in such rigid straight lines. In actual tissues, cells naturally disperse in all directions within a continuous three‐dimensional space. Therefore, the cell distribution obtained through this interpolation contradicts biological tissue organization.

To bypass artificial cell arrangements and sample destruction, researchers explored predicting three‐dimensional ST data directly from three‐dimensional tissue images. The VORTEX framework [196] represents a prominent example of this predictive approach. It utilizes non‐destructive 3D imaging techniques to capture the complete morphology of an intact tissue block. Researchers then physically sequence only a few sparse sets of 2D slices from that specific tissue block. Finally, VORTEX uses deep learning algorithms to match the continuous 3D morphological features with the sparse 2D molecular data. This predictive imaging approach provides specific biological advantages. It completely bypasses exhaustive serial sectioning and preserves the structural integrity of clinical samples. More importantly, this approach addresses the microscopic structural flaws of mathematical interpolation. VORTEX assigns molecular profiles directly to the actual 3D physical morphology of the intact tissue. It does not force artificial connections between discrete 2D slices. Consequently, the cellular distribution within this predicted 3D dataset aligns more closely with continuous tissue architecture. Nevertheless, this prediction paradigm has notable computational constraints. Its performance hinges heavily on solid biological correlations between tissue morphology and gene expression. In practice, many pivotal genes exhibit dramatic expression shifts that are not accompanied by any visible morphological variations in tissues. Such prediction algorithms are fundamentally unable to capture the spatial variability of these specific genes. This biological constraint currently limits the broader application of morphology‐based predictions.

Current technologies encounter limitations in reconstructing 3D spatial omics data. Direct physical 3D sequencing faces limitations such as insufficient penetration depth and high cost, and computational methods also have certain technical shortcomings. Therefore, constructing robust 3D spatial models remains a significant scientific challenge. Stacking methods leave numerous physical gaps. Mathematical interpolation produces unnatural cellular structures. Imaging‐based predictions rely heavily on morphological correlations. A single method is insufficient to overcome these obstacles. To construct dense 3D datasets, researchers must fundamentally integrate advanced experimental hardware with sophisticated computational algorithms. Researchers need to extract deeper, more continuous physical measurements directly from intact tissues. Simultaneously, advanced algorithms must perform mathematical inferences on the remaining unmeasured space with high biological accuracy. This synergistic integration of physical experiments, chemical techniques, and computational modeling is the ultimate solution. This combined approach aims to ultimately address the dimensionality gap problem and construct dense and continuous 3D spatial omics data [197].

### Virtual cell modeling

Historically, researchers have typically employed targeted gene screening and high‐throughput drug testing in functional genomics research. However, these traditional physical methods face cost pressures, including economic and human resource constraints. To overcome these physical and economic bottlenecks, researchers utilize computational modeling to construct digital models known as virtual cells [198] (Figure 4C). A virtual cell is a computational model that maps cellular states into a mathematical latent space, enabling accurate simulation of cellular structure, dynamic responses, and downstream functional changes induced by external perturbations. These computational models can accurately simulate cellular structure and dynamic response mechanisms. In a prospective view, researchers could potentially use mathematical methods to identify drug toxicity and efficacy deficiencies prior to physical experiments. The primary goal of virtual cells is to accurately predict downstream functional changes caused by external perturbations. Typical perturbations include targeted gene knockout or the introduction of novel therapeutic compounds. Researchers can mathematically link observed phenotypes to specific cellular signaling pathways. This mathematical tracing generates verifiable hypotheses for specific drug mechanisms.

The difficulty in constructing a virtual cell model lies in mapping complex cellular states into a highly compressed mathematical latent space. Currently, researchers have developed large‐scale single‐cell foundation models to construct these necessary mathematical representations. For example, the Geneformer and scGPT algorithms operate as foundational encoders [199]. These models learn general statistical rules from millions of undisturbed single‐cell transcriptomes to compress high‐dimensional gene expression data into abstract mathematical embeddings. Within these established latent spaces, the algorithms use purely mathematical vector algebra to model biological perturbations. PerturbNet [200] framework implements perturbation prediction based on this idea: first, the baseline cell features without perturbation are mapped to an abstract latent space, then various external perturbations are mathematically encoded as independent perturbation vectors. Subsequently, the perturbation vectors are directly applied to the embedding representation of the baseline cells, and finally, a generative decoder reconstructs the complete post‐perturbation transcriptomic profile. This purely data‐driven generative model has zero‐shot prediction capability, allowing direct inference of single‐cell responses to unknown small molecules or gene mutations based on compound chemical structures or gene function annotations. Currently, studies have used PerturbNet [200] to predict the complex phenotypic effects of specific point mutations in the GATA1 gene, identifying mutation sites that alter hematopoietic stem cell differentiation. In terms of biological validation, the computational predictions of this method align with physical DNA‐binding domains at the known protein structure level. Despite these capabilities, this data‐driven model lacks explicit biological constraints. Therefore, these unconstrained deep learning architectures often generate transcriptome predictions that contradict biological principles.

To address this specific limitation, researchers have developed mechanism‐constrained predictive models. This computational paradigm integrates existing biological prior knowledge into deep learning architectures to mitigate biological inconsistencies. The GREmLN framework implements this specific constrained paradigm. Standard unconstrained neural networks typically calculate generalized associations across the entire transcriptome. In contrast, GREmLN [201] explicitly embeds known gene regulatory networks directly into its computational architecture. These predefined biological networks function as rigid mathematical constraints. The algorithm utilizes Chebyshev polynomials to mathematically simulate signal diffusion strictly across these defined biological pathways [202]. This mechanism restricts the computational attention of the neural network. It forces the deep learning architecture to focus exclusively on biologically credible gene interactions. This mechanism‐driven approach provides a distinct analytical advantage. It successfully prevents the generation of non‐physical transcriptomic profiles. By strictly adhering to known biological rules, models like GREmLN execute complex analytical tasks. For example, the algorithm identifies specific master regulators driving cellular differences between healthy and diseased states. Furthermore, this highly constrained search space requires significantly fewer training parameters and data samples compared to unconstrained black‐box models. Despite these structural constraints, mechanism‐constrained single‐cell models contain a spatial limitation. These specific computational frameworks simulate single cells in isolated environments. However, specific cellular responses are profoundly dependent on their inherent spatial microenvironment. This local microenvironment contains complex intercellular communication and strict physical boundaries. Purely single‐cell predictive models typically fail to accurately predict systemic tissue responses. They completely ignore paracrine signaling and specialized spatial microenvironments. Therefore, researchers must develop algorithms with spatial awareness capabilities that leverage omics data to simulate real tissue responses. Otherwise, the resulting biological conclusions will only exist in scenarios where they are impossible.

Constructing these advanced spatial models requires massive amounts of specific perturbation data for training. However, standard spatial transcriptomics inherently destroys the biological tissue during sequencing. Researchers cannot sequence a physical tissue slice, apply a specific drug, and subsequently sequence that exact same slice. To overcome this measurement constraint, researchers utilize in situ spatial functional genomics. Experimental platforms like Spatial Perturb‐Seq perform sparse genetic editing directly within intact tissues [202]. This specific strategy mutates only a minute fraction of cells. These edited cells remain entirely surrounded by unedited normal cells. This specific spatial configuration provides the essential mathematical contrast for predictive algorithms. It explicitly decouples the direct transcriptomic shift of the edited cell from the paracrine influence exerted on surrounding normal neighbors.

Supported by these native spatial omics datasets, algorithms mathematically simulate the physical propagation of biological interventions. The SpatialProp framework executes spatial topology modeling. This algorithm conceptualizes the tissue microenvironment as an interconnected physical graph and models it using graph neural networks. The model mathematically calculates how a highly localized intervention physically diffuses across the tissue architecture. For example, it computes the downstream spatial effects of a single‐cell gene knockout on distant cellular neighbors [203]. Through continuous mathematical updates, SpatialProp [203] maps the cascade responses of specific local perturbations and generates prediction maps of whole‐tissue responses.

Furthermore, advanced generative architectures simulate complex spatial tissue responses under extreme environmental perturbations. The Squidiff algorithm [204] utilizes a conditional denoising diffusion implicit model to execute this spatial simulation. This specific framework integrates a continuous diffusion process with a specialized semantic encoder. The semantic encoder mathematically maps initial single‐cell transcriptomic profiles into a unified abstract latent space. Within this specific space, the algorithm encodes external biological stimuli as distinct mathematical direction vectors. The model executes latent manipulation through mathematical vector addition and linear interpolation. Subsequently, the conditional diffusion process systematically denoises pure Gaussian noise based entirely on these modified semantic variables. This inverse denoising mechanism synthesizes high‐resolution transcriptome maps of the perturbed tissue microenvironment. This generative approach predicts complex spatial responses to external stimuli.

However, spatial topological correlations are not equivalent to causal response mechanisms. Advanced spatial models must explicitly decouple intrinsic cellular regulatory mechanisms from external environmental signals. For example, the Celcomen model [205] utilizes rigorous causal reasoning to achieve this specific mathematical decoupling. This algorithm mathematically separates intracellular gene interactions from intercellular environmental influences. This rigorous causal separation enables advanced counterfactual reasoning in spatial microenvironments. Researchers use the Celcomen model to simulate complex hypothetical biological scenarios. For example, the algorithm predicts dynamic transcriptomic responses in surrounding cancer cells after computationally removing specific immune cell types from tissue. The introduction of causal reasoning transitions spatial models from correlational networks to mechanistic tissue simulators.

Although PerturbNet, GREmLN, SpatialProp, Squidiff, and Celcomen have different research focuses (data‐driven/mechanism‐constrained, Single‐cell/spatial dimension, association analysis/causal inference), they can all simulate perturbation responses at the cellular or tissue level. In contrast, foundational encoders like Geneformer and scGPT are not virtual cells themselves but are core tools for constructing latent space representations.

Different virtual cell algorithms present specific computational trade‐offs. Future development targets should focus on interpretable spatial modeling. Current predictive architectures typically function as opaque computational black boxes, lacking integration of specific biological mechanisms. Therefore, in the future, researchers must seamlessly integrate the spatial cellular environment with strict biophysical constraints and directly embed established physical and biological laws into core artificial intelligence algorithms. The primary objective of virtual cell engineering is direct clinical application. Researchers aim to utilize these advanced computational agents to execute large‐scale in silico clinical trials in the future. Ideally, these frameworks would perform counterfactual reasoning on virtual patient models to evaluate combination therapies.

### Summary

Systems biology aims to integrate structural reconstruction and predictive modeling. Static 3D anatomical atlases are merely observational snapshots of cellular structure, inherently lacking dynamic functional responses. Conversely, isolated virtual cells lack necessary physical boundary constraints. They exclude mechanical forces and local structural signals. Therefore, researchers must firmly anchor predictive computational models within the cell's inherent 3D topological coordinate system. This mathematical integration links intracellular molecular logic with extracellular physical geometry. It transitions isolated predictive algorithms into spatial tissue models.

Currently, there are numerous types of virtual cell technologies. However, life phenomena cannot be interpreted simply through mathematical modeling. Therefore, how to integrate spatial omics data, cell morphology data, and other spatial information to build robust virtual cell models remains a major challenge for bioinformatic researchers. In conclusion, future modeling frameworks must integrate data‐driven inferences with established biological knowledge. This integration drives the transition of life sciences toward in silico functional interpretation.

## TEMPORAL MODELING OF SPATIAL OMICS

### Introduction

**Figure 5:**
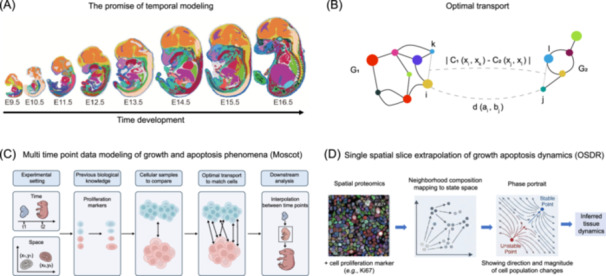
Spatial omics temporal modeling. (A) The promise of temporal modeling in spatial omics is to construct a comprehensive atlas of dynamic biological changes across the temporal dimension. (B) Core algorithm for multi‐time‐point slice alignment: Optimal transport. (C) Multi‐time point slice alignment: taking moscot as an example, based on multi‐time point spatial transcriptomic data and combined with prior information of growth and apoptosis‐related genes, the unbalanced optimal transport algorithm is applied to realize temporal slice alignment, thereby providing a foundation for cell lineage tracing. (D) Single‐slice modeling algorithm for growth and apoptosis: taking OSDR as an example, spatial proteomic snapshot data combined with prior gene sets related to growth and apoptosis are used to construct a state phase diagram, enabling the prediction of growth and apoptosis dynamics in cell populations.

The spatial significance of spatial omics is undeniable, but the concept of time is equally crucial [206]. Although technological flux and resolution continue to improve, most spatial omics techniques are based on snapshots and are destructive. Therefore, the time dimension of development, disease progression, and treatment response cannot be directly observed [207, 208]. Based on this, we must calculate the reconstruction time information. But this is not simply alignment integration, we need to consider changes in quantity, features, and interpretation of biological phenomena. Even the same type of cells can undergo significant changes in cell morphology and molecular state between different time points in different sections. This section focuses on temporal modeling [209, 210]. The promise of temporal modeling is that it allows researchers to clearly capture the spatiotemporal trajectories of organisms (Figure 5A).

The temporal modeling of spatial omics data can be understood from two perspectives: integrating temporal spatial data (multiple time points) or inferring dynamics from a single slice. The core of both strategies is to jointly model spatial omics information with growth and apoptosis rates or velocity fields.

### Multi‐time‐point data alignment for dynamic processes of cell growth and apoptosis

The core goal of time comparison is to achieve precise matching between tissue regions and cell populations in tissue snapshots of different developmental stages or disease processes, which requires handling dynamic changes in cell numbers caused by growth, apoptosis, and migration. The Optimal Transport (OT) algorithm (Figure 5B) is widely used in the task of comparing spatial omics data [211]. The core principle is to combine transcriptome similarity with spatial structural features by calculating the probability coupling between cell distributions [212]. Unlike traditional one‐to‐one matching models, OT coupling enables many‐to‐many matching correspondence, which is more aligned with the biological characteristic of cells collaborating in populations to perform physiological functions and transmit signals. In cross temporal comparison tasks such as development, disease progression, or tissue regeneration, it is necessary to consider changes in cell abundance caused by cell proliferation and apoptosis [213]. Furthermore, unbalanced OT [214] variants allow relaxation of the mass conservation constraint. In other words, they permit changes in total cell count, thereby better reflecting tissue growth and cellular population dynamics [215]. The other two alignment strategies are registration‐based alignment and trajectory‐aware alignment: the former relies on the absence of significant mutations in the spatial structure of multi‐time‐point data, while the latter is based on the core assumption of temporal smoothness.

Researchers should recognize that all four alignment approaches mentioned above have inherent methodological limitations (Table 4): balanced OT fails in the presence of cell death, proliferation, or regional changes in cell abundance; unbalanced OT tends to overinterpret noise as genuine cell migration and performs unstably on extremely sparse and low‐quality data; registration‐based alignment assumes that the spatial structure across time points remains unchanged, thus failing under severe tissue morphological alterations, folding, sectioning artifacts, or rapid structural remodeling; trajectory‐aware alignment relies on temporal smoothness, so abrupt data changes at any time point can lead to erroneous alignment. Overall, unbalanced OT is preferred for modeling cell growth and apoptosis when data quality is high; registration‐based methods can be adopted if tissue morphology shows no significant variation; and trajectory‐aware alignment may be considered when the dataset lacks early‐stage abrupt changes.

**Table 4:** Horizontal comparison of the core algorithms for multi‐time‐point spatial data alignment.

Method	Key assumptions	Where the method breaks down
Balanced OT	Mass conservation; total cell abundance/transcript count is unchanged over time	Fails when cell death, proliferation, or regional cell loss/gain occurs; cannot handle unbalanced tissues
Unbalanced OT	Allows mass creation/destruction; weak cost consistency; no strict mass balance	May over‐interpret noise as true cell movement; unstable under extremely sparse/low‐quality data
Registration‐based alignment	Spatial structure continuity; smooth deformation field; no drastic tissue reshaping	Breaks under large morphological changes, tissue folding, cutting artifacts, or rapid structural remodeling
Trajectory‐aware alignment	Temporal smoothness; cells follow continuous differentiation/transition paths	Fails for discontinuous cell state transitions, non‐sequential time points, or non‐directional spatial remodeling

For serial section reconstruction, balanced approaches such as PASTE apply Fused Gromov‐Wasserstein distance to match both expression similarity and spatial structure [216]. These methods handle platform variability and cases where sections only partially overlap. They have become widely adopted in tissue atlasing and multi‐section integration [40, 51, 217]. For the scenario of aligning multiple sets of temporal data with time dimension, different non‐equilibrium OT methods demonstrate unique advantages. For example, moscot [218] achieves precise alignment of multiple sets of temporal data by fusing prior information of growth‐ and apoptosis‐related genes (Figure 5C). This method has been validated on a mouse embryo dataset containing 500,000 cells. DeST‐OT is also based on the non‐equilibrium OT framework, but does not rely on prior genetic information. Instead, through the design of a triple objective function, it models the direct correspondence between a single pair of cells, the ancestor‐descendant type of cell branching evolution relationship, and the complex correspondence pattern between multiple pairs of cells. Its effectiveness has been validated in data related to the axolotl. In addition, DeST‐OT has innovatively introduced a quantitative method for spatially specific growth and apoptosis rates, which helps researchers quickly locate tissue functional regions and explore key genes related to growth and apoptosis processes, further expanding the application value of spatiotemporal dynamic analysis [219].

### Single spatial slice extrapolation of growth apoptosis dynamics

This type of method only requires one slice to calculate the dynamic changes of cells directly on the slice. The representative method for constructing temporal dynamic models based on single tissue slices is OSDR [220, 221, 222] and stVCR [223].

OSDR breaks through the limitations of traditional human tissue biopsies that can only provide static snapshots and cannot track the dynamic changes of cell populations over time (Figure 5D). This method is based on spatial proteomics data and Ki67 cell division markers, and constructs a dynamic model capable of inferring temporal changes in tissue cells by analyzing the regulatory effect of neighborhood composition on division rate. In breast cancer samples, OSDR successfully reproduced the thermal/cold fibrosis bistability formed by the interaction between fibroblasts and macrophages. OSDR also found the excitable impulse immune response circuit composed of T cells and B cells. At the same time, this method can accurately predict the collapse of the tumor cell population from treatment responders only through early treatment biopsy of triple‐negative breast cancer patients, while non responders remain stable. These results are robust and reliable in multiple independent cohorts, providing a new technical path for using a single clinical biopsy to analyze tissue dynamics, reveal TME mechanisms, and early predict treatment effects.

stVCR constructs joint models based on spatial omics, RNA velocity analysis [224, 225], partial differential equations (PDEs), and neural networks. It adopts spatiotemporal continuity equations to accurately characterize the dynamic patterns of cell migration, proliferation, and apoptosis. This method can not only analyze the growth and apoptotic properties of various cell types, but also deduce spatial migration vector fields to achieve quantitative characterization of cellular dynamics. To address the common challenge of sparse sampling time points in practical experiments, it provides flexible interfaces for integrating prior biological knowledge. The intermediate states it produces exhibit superior spatiotemporal continuity and effectively prevent unphysical cell jumps and trajectory discontinuities. Validated on datasets of injured axolotl brain regeneration, stVCR successfully identifies directed cell migration and elevated proliferation rates around wound spots. It also accurately screens migration‐driving and growth‐driving genes, which offers solid methodological support for further exploring the regulatory mechanisms underlying brain regeneration.

### Benchmarking, challenges, and future frontiers

Evaluating spatiotemporal alignment is difficult because ground truth is rarely available. Additionally, many ST assays destroy tissue as they measure, making verification measurements against the original tissue impossible. Furthermore, time‐series samples typically come from different individuals, introducing biological variation that complicates comparison. Finally, tissue geometry and cell composition continuously change over time. Because direct validation is not possible, evaluation must instead rely on measures of biological plausibility. These include checking that aligned anatomies are coherent, that marker genes localize to expected tissues, and that measurements are consistent when repeated independently. One also needs temporal plausibility metrics that can distinguish growth‐related changes in tissue shape from cell movement. A major limitation is that the field currently lacks shared benchmarking standards. This makes it difficult to fairly compare different methods on equal footing.

As spatial omics technologies improve, computational speed has become a limiting practical problem. Modern platforms such as CosMx, Stereo‐seq, Visium HD, and Xenium collect data at very high resolution and volume. As such, aligning such large datasets using simple pairwise comparisons becomes computationally infeasible. To reduce computation time, researchers have developed various approximations. Hierarchical alignment, sparse registration, and low‐rank approximations of optimal transport couplings are examples. These speed up calculations but sacrifice some alignment accuracy [226, 227]. Recent advances using streaming algorithms and factorized optimal transport provide some improvement [228, 229, 230], but a fundamental tension remains: increasing computational efficiency typically means reducing alignment accuracy. This trade‐off has not yet been resolved.

Comprehensive four‐dimensional tissue modeling requires moving beyond current methods that align sections pairwise. Instead, novel approaches should align point clouds in three and four dimensions, using all time points jointly rather than sequentially. Such methods must explicitly represent growth, cell movement, and tissue remodeling. Achieving this will necessitate close collaboration among computational biologists, imaging specialists, and developmental biologists with expertise in tissue biology.

Beyond scalability, temporal uncertainty and differences in measurement types add complexity. Taking sequential measurements from the same tissue is logistically difficult because each measurement destroys the tissue. This destructive sampling makes it hard to distinguish between real biological change and measurement artifacts. The problem worsens when different assays are used at different time points. Multimodal optimal transport methods and learned representations can partially address this mismatch, but the field still lacks standardized, production‐ready pipelines for cross‐modality temporal alignment [231]. This limitation constrains efforts to build comprehensive atlases and to model disease.

In the future, foundation models may help address feature heterogeneity. Pretrained embeddings from models such as scGPT, scBERT, and Geneformer can be more robust to technical noise [232, 233]. These representations could support several applications: defining alignment costs, smoothing state transitions, and imputing missing time points. However, there are significant risks. Powerful models may inadvertently remove real biological change if temporal variation and batch effects overlap and confuse the model. This could lead to incorrect alignments where developmentally distinct populations appear matched when they should not. Additionally, models trained on healthy tissue may not accurately represent disease states. Encouragingly, although live‐cell imaging and lineage tracing are still under development, advances in probe chemistry, imaging speed, and phototoxicity mitigation enable these techniques to serve as a promising gold standard for model validation.

## APPLICATIONS OF SPATIAL OMICS IN MEDICAL RESEARCH

**Figure 6:**
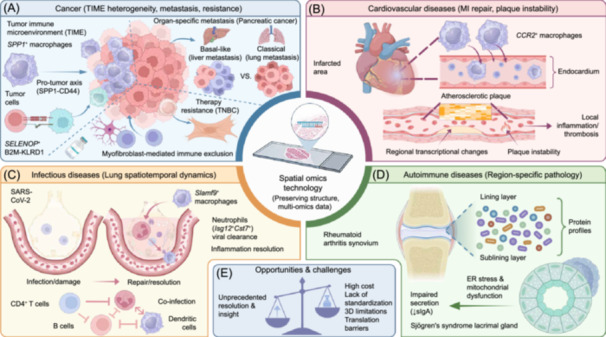
Applications and challenges of spatial omics in medical research. Spatial omics technologies preserve tissue spatial architecture and integrate multi‐omics data, offering high‐resolution insights into the mechanisms underlying a broad spectrum of diseases. (A) Cancer research: Deciphering tumor immune microenvironment (TIME) heterogeneity, revealing SPP1^+^ macrophage‐ and myofibroblast‐mediated immune exclusion, as well as TGFB1^+^ cell‐driven therapeutic resistance, and identifying phenotypic differences in organ‐specific metastasis of pancreatic cancer. (B) Cardiovascular diseases: capturing the repair dynamics of CCR2^+^ macrophages in myocardial infarction regions and transcriptomic changes in atherosclerotic plaques, and elucidating the mechanisms of plaque instability, inflammation, and thrombosis. (C) Infectious diseases: Using SARS‐CoV‐2 infection as an example, tracking the spatiotemporal dynamics of the lung during infection‐repair processes, and dissecting the roles of Slamf9^+^ macrophages and neutrophils in viral clearance and inflammation resolution. (D) Autoimmune diseases: Uncovering region‐specific pathological features in the synovium of rheumatoid arthritis and the lacrimal glands of Sjögren's syndrome, and elucidating the molecular mechanisms underlying impaired secretory function. (E) Opportunities and challenges: spatial omics provides unprecedented resolution for disease research, while facing challenges including high cost, lack of standardized protocols, limitations in three‐dimensional tissue analysis, and barriers to clinical translation.

At present, spatial omics has played a breakthrough role in the medical field [234, 235, 236]. Its core value lies in breaking the limitation of traditional research that is difficult to capture the cell space interaction and molecular characteristics. By accurately mapping the cell composition, molecular expression spectrum and intercellular regulatory network of the tissue microenvironment, it provides unprecedented resolution and panoramic perspective for the analysis of the pathogenesis of major diseases such as cancer [237, 238], cardiovascular disease (CVD) [239], infectious diseases [240], and autoimmune disease [241, 242] (Figure 6A). This section presents the current research and findings of spatial omics for specific diseases.

### The application of spatial omics in cancer research

Analyzing the composition and spatial organization of the tumor immune microenvironment is crucial for understanding cancer progression and treatment. Spatial omics is commonly used to map the TME [243, 244], revealing the spatial distribution of tumor molecular subtypes and prognostic marker expression and interactions across multiple cell types. It can be seen that spatial omics is crucial for understanding how TME assists tumor evolution, metastasis, and immune escape, as well as identifying potential therapeutic targets.

Lindsay et al. conducted a comprehensive and systematic spatial proteomic analysis of 14 types of cancer in 2019 patients using multiplex immunofluorescence (mIF) technology, and identified 5 conserved TIME spatial factors across cancer types: PC1−PC5 [245]. These 5 factors are highly consistent in early and late‐stage tumors of different cancer types and belong to the universal characteristics of solid tumor TIME. A systematic understanding has been established: cancer type and stage explain <20% of factor variations, with most variations driven by the intrinsic immune features of the tumor. By integrating these five factors, researchers established a tumor immune microenvironment score that enables significant stratification of patient prognosis.

Currently, there are many studies emerging on various types of cancer. Qiu et al. drew a single‐cell spatial protein map of Hepatocellular Carcinoma (HCC), analyzed the spatial heterogeneity between HCC tumors, established a prognostic related spatial pattern for stem cell carcinoma, and integrated multiple omics and in vitro experiments to reveal the important finding that the spatial interaction between *Vimentin* overexpressing macrophages and Treg cells promotes the disease progression of HCC [246]. de Oliveira et al. used Visium HD to create single‐cell level high‐resolution maps of the immune microenvironment in colorectal cancer (CRC) [247]. Through the 8 μm resolution data of Visium HD, the team identified two functionally distinct subpopulations of macrophages: *SPP1^+^* macrophages and *SELENOP^+^* macrophages. The two are not only spatially separated, but also have completely different functional pathways: the *SPP1^+^* subgroup is enriched near *TGFBI^+^/PERP^+^* tumor cells, activating coagulation, cholesterol metabolism, and KRAS signaling pathways, which may promote tumor progression and inhibit immune surveillance through the SPP1–CD44 axis. The SELENO*^+^* subgroup is adjacent to *REG1A^+^/LCN2^+^* tumor cells, driving inflammatory responses and complement system activation through the NF‐κB pathway, and regulating T cell metabolism through the B2M–KLRD1 axis. Anirban Maitra's team revealed that the transcriptome status of pancreatic cancer cells had significantly changed when they were transferred from the primary location to a specific organ, especially between liver and lung metastases. Compared with lung metastases [248], liver metastases have more abundant basal‐like cell states, while classical cell states are more common in lung metastases. This difference may indicate that PDAC undergoes preferential cell line transformation during different organ metastasis processes. The study also revealed the spatial distribution characteristics of immune and stromal cells in the TME: myofibroblasts (myCAFs) expressing *TGFβ1* were significantly enriched in tumor regions associated with basal‐like cell states, and these cells often formed fibrous cell bands surrounding tumor cells. In tumor regions associated with basal‐like cell states, the expression of *CXCL12* was associated with plasma cell rejection, which may weaken local humoral immune responses. There are many similar studies on single cancer species. For example, Mardamshina et al. found that the heterogeneity of protein expression in high‐grade breast cancer tumors is higher by using multi‐region spatial proteomics, and this heterogeneity is more affected by TME than genomic variation [249]. Zhang et al. comprehensively used single‐cell sequencing, 10x Genomics Visium HD and other cutting‐edge technologies to accurately reveal for the first time that the sensory nerve in the tumor is the “culprit” leading to the immunotherapy resistance of some triple‐negative breast cancer patients, and found that migraine drugs can reverse the above process and synergize with immunotherapy, providing a potential new strategy for overcoming the immunotherapy resistance of triple‐negative breast cancer [250]. Wei et al. used single‐cell technology combined with spatial proteomics and transcriptomics to analyze patient samples before and after drug treatment, and found that drug‐resistant tumors formed a spatially structured immune suppression network (PI3K–AKT–mTOR sustained activation + immune rejection), making it difficult for drugs to act on them [251]. These studies indicate that spatial omics technology has become a powerful scientific research weapon for analyzing TMEs [252], exploring cancer‐related mechanisms [253], and promoting patient treatment.

### The application of CVD research

CVD is the deadliest disease category globally, and its pathological process is highly dependent on the complex spatial interactions between cell types, molecular states, and tissue microenvironments. In recent years, spatial omics technology provides unprecedented resolution for analyzing the pathogenesis of CVDs such as myocardial infarction (MI), heart failure, and atherosclerosis by synchronously obtaining high‐dimensional data of transcriptome, epigenome, proteome, and even metabolome on the premise of preserving the original structure of tissues.

The tissue repair after MI [254] highly relies on timely infiltration and functional coordination of immune cells. The traditional view is that myeloid cells mainly enter the injury area through microvascular extravasation in the infarct margin, but there is a lack of systematic evidence on whether the endocardium is involved in immune recruitment. Due to the lack of high‐resolution techniques for in situ analysis of molecular, protein, and cellular interactions, the role of the endocardial microenvironment in the acute phase of MI has long been overlooked. Wünnemann et al. used spatial omics techniques to explore a novel mechanism by which myeloid cells (especially *CCR2^+^* monocytes/macrophages) can attach to and infiltrate the infarcted area through the endocardium, reaching a peak infiltration within 24 h [255] (Figure 6B).

Coronary atherosclerotic plaque is the main pathological basis leading to acute coronary syndrome (ACS). For a long time, plaque instability has been considered as the key factor leading to acute events. Traditionally, plaques that are prone to ACS are often believed to be caused by unstable cellular structures surrounding them. However, the emergence of spatial omics technology has revealed more potential mechanisms of plaque instability, such as the transdifferentiation of specific cells, activation of local pro‐inflammatory and thrombotic signals, and so forth. Gastanadui et al. used GeoMx spatial analysis platform and full transcriptome map to reveal significant cell specific and regional transcriptional changes in coronary atherosclerotic plaque [256]. These changes not only indicate a high degree of heterogeneity in unstable plaques but also demonstrate that activation of local inflammation and thrombus formation signals may be important mechanisms leading to plaque instability.

### The application of spatial omics in infectious diseases research

The pathological core of infectious diseases lies in the spatio‐temporal game between pathogen and host immune system. The dynamic process of recruitment, activation, functional coordination, and tissue repair of immune cells all depend on precise spatial regulation and molecular interaction. This section focuses on lung research to explain the application of spatiomics in infectious diseases.

The lungs are organs that are continuously exposed to external stimuli, and their immune monitoring function is crucial for quickly and effectively responding to pathogen stimuli to activate host immune responses. Therefore, it is crucial to explore how lung immune cells dynamically coordinate time and space to respond to infections, clear pathogens, reduce inflammation, and restore homeostasis. The Xuetao Cao team established a model of SARS‐CoV‐2 infection in Syrian hamsters to investigate the cellular and molecular spatiotemporal dynamics during the entire process of lung virus infection and clearance (Figure 6C). Single‐ cell, spatial transcriptome, and immune mapping techniques were used to map the spatiotemporal dynamics of SARS‐CoV‐2 lung tissue in Syrian hamsters before infection (d0), during acute tissue injury (d2), during immune response (d5), during virus clearance (d7), and during inflammation resolution and tissue repair (d14) [257]. During the research process, researchers discovered a group of *Slamf9^+^* macrophages derived from monocytes, which were induced to produce after SARS‐CoV‐2 infection and were able to resist the damage caused by SARS‐CoV‐2. *Slamf9^+^* macrophages contain SARS‐CoV‐2 and recruit and interact with *Isg12^+^Cst7^+^* neutrophils to clear the virus. After virus clearance, *Slamf*9*^+^* macrophages differentiate into *Trem2^+^* and *Fbp1^+^* macrophages, promoting the resolution of later inflammation and ultimately supplementing alveolar macrophages. Double infection of lung influenza related bacteria will lead to an increase in incidence rate and mortality. The John F. Alcorn team used Visium technology to systematically analyze the synergistic mode of immune cells during the process of double infection [258]. Researchers have found that during infection, the recruitment of neutrophils and interstitial macrophages from lung parenchyma to the airway is inhibited, which may affect pathogen clearance, and secondary bacterial double infection can disrupt the interactions between CD4^+^ T cells, B cells, and dendritic cells. This significant discovery provides a theoretical basis for elucidating the abnormal inflammatory mechanisms of double infected lungs and also contributes to the development of therapeutic drugs targeting key immune cell recruitment pathways.

### The application of spatial omics in autoimmune diseases research

The essence of autoimmune diseases is the abnormal attack of the immune system on one's own tissues, and their pathological characteristics highly depend on the local cellular composition, molecular microenvironment, and spatial interactions between cells in the diseased tissue. Traditional batch detection techniques are difficult to accurately capture this region‐specific pathological change. At present, spatial omics technology has been widely applied. Wang et al. used laser capture microdissection (LCM) combined with mass spectrometry based proteomics technology to analyze the pathological microenvironment regions of rheumatoid arthritis synovial tissue [259]. Compared with healthy controls, they successfully obtained protein expression profiles of the lining layer and sub‐lining layer of the affected synovium. Research has found that key pathogenic proteins of rheumatoid arthritis, including membrane proteins and extracellular matrix proteins, exhibit differential expression patterns in different regions of the affected synovium, as well as enrichment characteristics of various types of cells, thereby screening potential protein targets for therapeutic interventions. This integrated analysis strategy can more comprehensively reveal the pathological characteristics of rheumatoid arthritis synovium, making up for the shortcomings of traditional batch transcriptomics in target discovery. SjD is the second most common rheumatic disease characterized by autoimmune pathological changes targeting the lacrimal gland that secretes tears, ultimately leading to chronic ocular surface disease. Researchers used spatial transcriptomics to analyze the expression profiles of lacrimal glands in wild‐type mice and platelet‐derived C‐reactive protein 1 deficient mice (spontaneous model of Sjogren's syndrome) [260] (Figure 6D). Research has found that the lacrimal glands of mice with Sjogren's syndrome exhibit endoplasmic reticulum stress in acinar cells, mitochondrial dysfunction in ductal epithelial cells, impaired secretion function of acinar and ductal epithelium, as well as gene expression patterns of myoepithelial cell contraction disorders accompanied by pro‐fibrotic remodeling. In addition, the expression of *Pigr* in acinar epithelium was significantly reduced (this receptor is responsible for transporting protective secretory IgA to tears), which was significantly correlated with the decrease in sIgA levels in mouse tears. These findings provide a theoretical basis for targeted epithelial cell and multicellular interaction therapy for autoimmune lesions in the lacrimal gland of Sjogren's syndrome.

### Opportunities and challenges

At present, spatial omics has pushed medical research into a new dimension, providing a new perspective for disease mechanisms and precise diagnosis and treatment. However, there are still many key challenges (Figure 6E) in moving towards clinical translation and large‐scale application: sequencing and chip technologies have limited resolution, while imaging technologies have slow imaging and are difficult to cover the entire transcriptome, making it difficult to support large‐scale clinical cohort studies. The existing methods rely heavily on thin tissue slices and cannot directly perform three‐dimensional in situ analysis on thick tissues or intact organs. At the same time, different clinical samples lack a universal process, with precise and complex equipment, high costs, and long cycles, making it difficult to meet the practical needs of large clinical pathology samples and fast turnover. Moreover, there is no unified clinical application standard and consensus in the industry.

## APPLICATIONS OF SPATIAL OMICS IN NEUROSCIENCE

The mammalian brain is a highly complex organ that relies on communication and collaboration among multiple neurons to achieve its neural functions [79, 261]. Since brain function is closely related to its spatial organization, the brain has become the main focus of current research in spatial omics [262]. This section will summarize the latest breakthroughs in spatial omics in the field of neuroscience and divide them into three parts: neural architecture and mechanisms [263, 264, 265, 266], neural development [267, 268], and neurological disorders [269, 270, 271].

### Spatial omics in neural architecture and mechanisms

Traditionally, the neuroanatomical understanding of the cerebral cortex relied primarily on Korbinian Brodmann's classical “cytoarchitecture” [272]. These physical boundaries are based on cell morphology and map the brain's overall structure. However, they could not distinguish the molecular differences hidden within similar cells, which ST can resolve. By mapping gene expression within its spatial context, ST redefines cellular identities and tissue boundaries at the molecular level. This approach not only creates accurate anatomical brain atlases [273, 274], but also reveals the molecular rules that govern neuronal arrangement, circuit wiring, and microenvironmental interactions.

#### Redrawing anatomical boundaries

**Figure 7:**
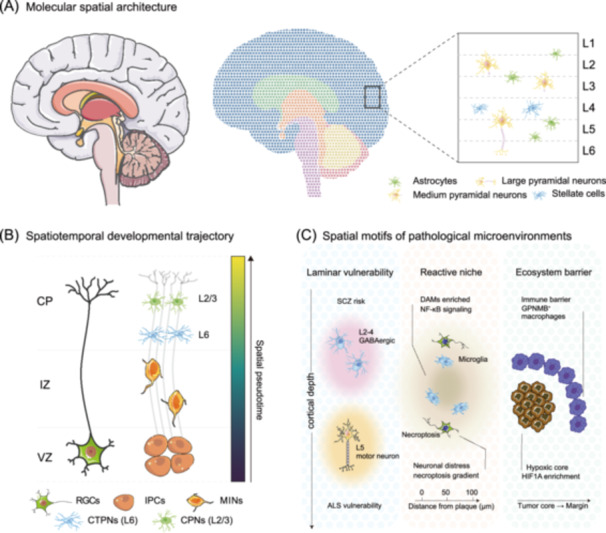
The applications of spatial omics in neuroscience. (A) Molecular spatial architecture. ST provides a molecular map of the mammalian neocortex. Compared with scRNA‐seq, ST gives more detailed information about the six‐layer (L1−L6) laminar boundaries across spacies, defined by specific cell types such as pyramidal neurons and astrocytes. This established a foundational reference for subsequent studies of developmental and pathological states. (B) Spatiotemporal developmental trajectory. ST reconstructs the dynamic cellular transitions during neocortical neurogenesis. Through bioinformatics analysis, the spatial pseudotime was also constructed for neurodevelopment. Precisely, they track the paths of progenitor cells over space and time: starting in the VZ, then moving to the IZ, and finally settling in the mature CP. (C) Spatial motifs of pathological microenvironments. Left: Laminar vulnerability. Risks of neurological disorders are distributed across different cortical regions. For example, SCZ risk is enriched in Layer 2−4 GABAergic interneurons13 and ALS risk is localized to layer 5 motor neurons. Middle, Reactive niche. In AD, Aβ plaques drive a distance‐dependent toxicity gradient (0–100 µm): from a DAM‐enriched core to outer regions of neuronal distress and necroptosis. Right, ecosystem barrier. In GBM, there is an ecosystem with an HIF1A‐enriched hypoxic core and a peripheral immunosuppressive barrier composed of GPNMB+ macrophages. Aβ, amyloid‐beta; AD, Alzheimer's disease; ALS, amyotrophic lateral sclerosis; CP, cortical plate; DAM, disease‐associated microglia; GBM, glioblastoma; IZ, intermediate zone; SCZ, schizophrenia; ST, spatial transcriptomics; VZ, ventricular zone.

The classical six‐layer (L1–L6) structure of the cerebral cortex is fundamental to understanding brain function. Early ST studies using 10x Visium successfully mapped gene expression patterns to these traditional layers [275]. However, many current spatial omics technology platforms have achieved single‐cell resolution and excellent capture efficiency. Driven by these high‐resolution tools, researchers can now identify spatial domains that are more refined and specific than traditional boundaries (Figure 7A).

This precise molecular mapping has now expanded to the whole‐brain scale across species, moving beyond static cell cataloging to redefining neuroanatomy [276, 277, 278]. In human studies, integrating ST with snRNA‐seq has produced high‐resolution atlases across 14 cortical regions, revealing the spatial distribution of more than a million individual cells. Beyond global architecture, this macro‐scale mapping also decodes the molecular logic of specific functional networks. For example, within human language hubs ST reveals major layer‐specific differences between the frontal and temporal cortices. This provides direct molecular evidence for the specialization of higher‐order brain areas beyond classical anatomy [279].

#### Mapping the spatial rules of the central nervous system (CNS)

Neurons in the CNS are not distributed randomly. Recent ST studies have revealed several key principles that shape this precise distribution.

First, at the macro‐scale of the cortex and cerebellum, cellular topography serves as a molecular blueprint for both functional hierarchy and evolutionary expansion. Within a single species, ST reveals continuous functional gradients. For instance, macaque Stereo‐seq atlases demonstrate that excitatory‐to‐inhibitory neuron ratios shift systematically from primary sensory regions to higher‐order association areas [280]. Comparing these maps across species further exposes the cellular basis of brain evolution. For example, primate‐specific neuronal subtypes in cortical layer 4—alongside specialized Purkinje cells in the cerebellum [281]—provide the molecular substrates for higher information processing capacity. Furthermore, ST tracking indicates that the deep cerebellar nuclei expanded during evolution through the repeated duplication of a basic cell type [282].

Second, lower CNS structures, such as the spinal cord, operate on a distinct and coordinate‐driven spatial logic. The fate and location of spinal neurons depend on their specific two‐dimensional signals. Indeed, high‐resolution ST captures a highly organized “sandwich‐like” microanatomy in the human spinal dorsal horn, featuring strictly alternating excitatory and inhibitory interneurons. Unlike the inside‐out radial migration of the cortex, this alternating lamination confirms that the axial coordinates determine cell identity in the spinal cord [268].

Finally, at the microscopic level, local cellular organization is governed by homotypic repulsion—“mosaic hypothesis” [283]. This principle suggests that neurons of the same type naturally avoid clustering together, forming an evenly spaced, non‐overlapping pattern which has been further proved by ST recently [284]. By keeping a consistent distance from identical neighbors, these cells can efficiently cover the entire tissue. This kind of spread model enables the brain to use its resources efficiently without waste.

#### Unveiling the multicellular microenvironment

The CNS is not only a network of neurons but a multicellular ecosystem. In addition to neurons, there are non‐neuronal cells, including astrocytes, microglia, oligodendrocytes, and vascular endothelial cells. These cells assemble into the specific “spatial niches,” which are essential for providing metabolic support, maintaining immune surveillance, and regulating synaptic transmission [285].

The transcriptional states of these non‐neuronal cells are always coupled to their neuronal neighbors. In the mouse somatosensory cortex, for example, the density and functional signature of microglia are determined by the identity of local excitatory neurons [286]. Specific microglia are enriched across different cortical layers, forming local ligand‐receptor networks with neighboring neurons. When researchers alter the identity of a deep‐layer neuron, nearby microglia will shift their states to adapt to the new microenvironment. With ST, detailed multicellular niches within CNS become achievable. It establishes the spatial framework required to understand how cells in the CNS are wired together during development and how these fragile microenvironments collapse in neurological disorders.

### Spatial omics in neurodevelopment

The assembly of the nervous system relies on a precise 4D framework in which the fate of neural progenitors is determined by their spatial position and time of birth [287]. Once their identity is set, these cells navigate to their locations to wire into functional networks. Spatial omics allow researchers to map the precise spatiotemporal trajectories of neurogenesis, revealing how cells decide their fates, navigate through tissue, and mature into different networks.

#### Patterning the spatial axes of the early nervous system

The architecture of the early nervous system is built on a precise spatial grid. Before neurogenesis, local morphogen gradients imprint spatial coordinates onto the early progenitors. ST now resolves this early chemical architecture, capturing how localized signals trigger the initial transcriptional divergence of neural lineages. Recent ST studies have mapped these early signals [288]. During early embryogenesis, such as in the human gastrula (Carnegie stage 8), 3D ST mapping precisely localizes the notochord and primitive streak, defining the molecular onset of neural induction. Following induction, the tissue folds into the neural tube, establishing the primary axes of the brain and spinal cord.

#### Mapping spatial pseudotime along the radial axis

The neocortex develops through a highly structured process known as inside‐out radial migration (Figure 7B). High‐resolution ST directly captures this spatiotemporal continuum. Recent cross‐species atlases have mapped the developing neocortex, tracing the stepwise transcriptional maturation of neurons as they ascend from VZ progenitors to fully specified CP neurons [289]. ST is also performed to localize the earliest‐born cells within their exact spatial lineage. These pioneer cells guide early cortical folding and circuit wiring [290]. Beyond in vivo tissues, ST profiling of brain organoids confirms that these in vitro models can recapitulate these natural spatial patterning rules [291].

Computationally, this physical migration provides a basis for measuring developmental time, a metric called spatial pseudotime. These tools calculate the speed and direction of cell maturation directly in the tissue. Such models provide definitive molecular proof for the classic ‘inside‐out’ migration pattern, confirming that a cell's physical position in the developing cortex serves as a direct readout of its precise developmental age.

#### Differentiating cell lineages across brain regions

While inside‐out radial migration defines neocortical assembly, the other brain regions follow diverse spatial rules [292]. By expanding mapping efforts across the entire CNS, ST now captures these diverse developmental lineages, uncovering both evolutionarily conserved trajectories and human‐specific specializations.

For example, in the hypothalamus, cross‐species ST identified a conserved core developmental program between mice and humans [293]. However, it also identified the unique progenitor zones in the human fetus that help give rise to more complex neuromodulatory networks. Similarly, within the developing cerebellum, ST traces the separate paths of Purkinje and granule cells, revealing the precise point of their divergence [294]. These atlases have also pinpointed novel neuroepithelial progenitors at the rhombic lip, which drive the expansion of the human cerebellum [295].

Beyond the brain, sensory systems and lower CNS follow similar strict spatial developmental rules. In the developing human retina, ST reveals when progenitors mature into photoreceptors and ganglion cells, and these data record the exact timing of retinal assembly [296]. Furthermore, in the spinal cord, ST reveals how the dorsoventral and mediolateral axes dictate cell fate.

#### Bridging molecular maps and cognitive networks

Neurodevelopment constructs the circuits necessary for cognition. ST bridges molecular profiles with macroscale function, aligning gene atlases with fMRI to reveal the biological basis of neural networks [297]. In the human cortex, gene transcription forms a spatial gradient from ventromedial to dorsolateral regions. These gradients match psychological functions, separating emotion from perception. This topography is dynamic, and its gradient sharpens from fetal stage to adulthood. As the brain matures, local gene expression synchronizes with global networks. Neurodevelopment is a long‐term process that calibrates and matures the molecular gradients that sustain human cognition [298].

### Spatial omics in neurological disorder

Brain diseases rarely damage tissue in a random manner. ST anchors gene expression back to the tissue's physical scaffold [299]. This high‐resolution mapping demonstrates that diverse brain disorders share recurrent spatial organization patterns. Synthesizing findings across human and model systems reveals that brain pathologies converge onto three recurrent spatial motifs: laminar vulnerability, reactive niches, and ecosystem barriers (Figure 7C) [300].

#### Laminar vulnerability

Selective vulnerability is a fundamental characteristic of brain diseases. Although risk factors like genetic mutations are often widespread, they selectively compromise specific cell types within defined anatomical layers [301]. ST directly visualizes this phenomenon, termed “laminar vulnerability,” which explains why certain brain layers are targeted while others are spared. In other words, pathological changes are preferentially concentrated in specific cortical layers (Figure 7C).

Neurodegenerative diseases often affect cells in specific locations. In amyotrophic lateral sclerosis (ALS), for example, spatial studies of the human motor cortex have identified gene expression changes concentrated in specific layers. A similar laminar vulnerability pattern is found in Alzheimer's disease (AD). For both sporadic and familial AD, early pathology is targeted in layers 4 and 5 (L4−5) of the middle temporal gyrus (MTG) [302]. This spatially restricted damage disrupts excitatory neurons and triggers a loss of local synaptic networks [303, 304]. This kind of vulnerability is not limited to the cortex or to adult life. In the subiculum, specific molecular patterns established early in development explain why the tau protein initially builds up in this precise area decades later in AD [305]. Expanding beyond the cortex, ST integrated with translatomic profiling in Parkinson's disease (PD) has pinpointed the exact spatial vulnerability of dopaminergic neurons in the substantia nigra [306]. Furthermore, mapping of the enteric nervous system reveals that alpha‐synuclein accumulation and dopaminergic degeneration occur synchronously in the colon myenteric plexus, validating the gut‐brain axis in PD [307].

#### Reactive niches

Pathological hallmarks such as amyloid plaques and tau tangles are not isolated. They recruit nearby non‐neuronal cells and then create “reactive niches.” This interaction alters the local transcriptome and establishes a gradient of stress and immune activation (Figure 7C) [308].

In AD [309], the physical distance of cells from β‐amyloid (Aβ) plaques is a key determinant of their phenotype: ST mapping shows that microglia and astrocytes within 50 μm of plaques mount a robust inflammatory response, while inflammatory signals decline sharply beyond this threshold. These plaque‐associated cells activate the NF‐κB signaling pathway and undergo profound immunometabolic reprogramming. Additionally, ST has uncovered the active regulatory role of neurons in inflammatory niche formation: during immunometabolic reprogramming, neurons act as local signaling hubs that accumulate DNA double‐strand breaks (DSBs) and activate adjacent microglia via antiviral‐like pathways [310], a complex intercellular crosstalk now directly visualized by in situ ST mapping.

This reactive niche architecture also governs other inflammatory and traumatic conditions. For example, in multiple sclerosis (MS), a distinct pathogenic zone is localized to the rim of chronic active lesions through ST mapping. This “lesion rim” is enriched for CD8*^+^* T cells and foamy microglia [311]. Interferon signaling promotes the accumulation of myelin‐derived cholesterol within these microglia. This impaired lipid efflux ultimately sustains persistent local tissue damage.

#### Ecosystem barriers

In this section, the application of spatial omics technologies to barriers in the neural ecosystem is discussed in the context of glioblastoma (GBM) research. GBM tumors have a highly structured microenvironment [312]. Within it, multicellular “oncostreams” and fibrotic networks form physical boundaries that promote local tumor invasion [313]. This spatial compartmentalization inherently limits drug penetration and helps malignant cells evade immune surveillance (Figure 7C) [314, 315].

GBM is highly compartmentalized, with a tumor core that is notably hypoxic and metabolically distinct from the rest of the tumor. ST mapping shows that this core is enriched with hypoxia‐inducible factors and contains specialized GBM‐associated fibroblasts (GAFs). By interacting with local myeloid‐derived cells through the COL6A3‐ITGA1 pathway, these GAFs induce profound vascular fibrosis. As a result, this rigid core functions as a mechanical barrier, directly blocking the delivery of chemotherapeutics. At the invasive tumor margin, ST identifies ‘oncostreams’, which are multicellular fascicles composed of spindle‐shaped cells. Modulated by the *COL1A1* gene, these spatial structures act as physical pathways for collective cell movement, enabling quick tumor growth into neighboring brain tissue. In addition to its structural compartments, GBM shows significant spatial metabolic heterogeneity. By integrating ST with 13C‐glucose mass spectrometry imaging, recent studies have revealed distinct intratumoral glycolytic and oxidative zones [316]. This spatial compartmentalization provides the structural molecular basis for the clinical failure of immune checkpoint inhibitors (ICIs) in recurrent GBM [317]. Spatial profiling of recurrent masses confirms that *PD‐1* blockade fails because the hypoxic core physically excludes effector T cells. At the same time, the tumor margins are enriched by specific *GPNMB^+^* macrophages, which create a localized immunosuppressive environment that neutralizes therapeutic efficacy.

Similar spatial compartmentalization occurs in other pediatric brain tumors. In Sonic hedgehog medulloblastoma, ST localizes tumor‐initiating progenitors to specific niches. Treatment with *CDK4/6* inhibitors induces the differentiation of these progenitors, which ultimately reduces the overall spatial heterogeneity of the tumor [318]. Meanwhile, spatial mapping identifies a stem‐like tumor population characterized by high midkine (MDK) expression, which establishes a localized immune‐suppressive microenvironment that contributes to rapamycin resistance [319].

### Conclusion and future perspectives

Spatial omics has redefined brain research, reinterpreting the brain as a highly coordinated network where architecture shapes function by anchoring gene expression to tissue's physical structure. It advances neuroscience in three core areas: cross‐species mapping of healthy brain molecular architecture, tracking spatiotemporal cell development and migration in the CNS, and uncovering distinct spatial patterns in neurological diseases. With the development of spatial omics, researchers' research in the field of neuroscience will become increasingly refined and scientific, better analyzing brain mechanisms [320, 321, 322].

## APPLICATIONS OF SPATIAL OMICS IN ANIMALS AND PLANTS

Spatial omics technology has not only played a huge role in neuroscience and medicine. In animals, it can accurately characterize cellular heterogeneity, developmental trajectories, and communication networks in development, disease, or other life processes. It provides a powerful tool for researchers in animal developmental biology, evolutionary biology, and other animal fields. In plant research, it allows the in situ dissection of spatial gene regulation and metabolic distribution underlying organogenesis, stress responses, and symbiotic interactions, providing key targets and theoretical support for crop improvement in stress resistance, quality enhancement, and molecular breeding. As a key technology bridging genotype and phenotype, spatial omics has advanced animal and plant life sciences into a new stage of spatiotemporal precision and systematic visualization [322]. This section focuses on both animal and plant research and elaborates the scientific discoveries and values of spatial omics in these two fields.

### Applications of spatial omics in animals

#### Gametogenesis and germ cell development

**Figure 8:**
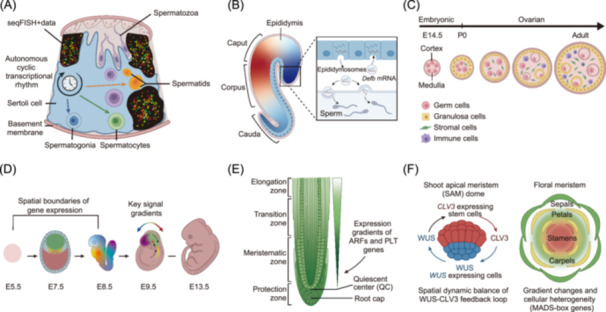
The applications of spatial omics in animals and plants. (A) Mouse epididymal region‐specific β‐defensin (Defb) expression gradient and directional mRNA transport via epididymosomes. Integration of single‐cell and spatial transcriptomics reveals that epididymosomes mediate directional transport of Defb mRNA from epididymal epithelium to sperm surface across the caput‐corpus‐cauda axis. (B) Mammalian ovarian spatiotemporal development and follicle fate regulation (mouse and pig models). Continuous spatiotemporal atlas from embryonic day 14.5 (E14.5) to adult reveals precise spatial arrangement of germ, granulosa, stromal, and immune cells coordinated by NOTCH signaling and extracellular matrix (ECM) proteins. (C) Mouse testicular seminiferous tubule spatiotemporal map by RNA sequencing combined with sequential fluorescence in situ hybridization (RNA seqFISH+). High‐resolution spatial transcriptomics reveals that Sertoli cells exhibit autonomous cyclic transcriptional rhythm and function as an active timer to synchronize gene expression programs with surrounding spermatogenic cells. (D) Mouse embryonic organogenesis three‐dimensional (3D) spatiotemporal map by Stereo‐seq (spatial enhanced resolution omics‐sequencing), Slide‐seq (slide‐based sequencing), and 10× Visium. Multi‐platform spatial transcriptomics generates continuous transcriptomic atlas from E5.5 to E13.5, revealing spatial boundaries during mesoderm induction, neural tube patterning, and organ primordia formation. (E) Arabidopsis root spatiotemporal development: multi‐omics analysis. Integration of spatial transcriptomics and metabolomics by matrix‐assisted laser desorption/ionization mass spectrometry imaging (MALDI‐MSI) and desorption electrospray ionization mass spectrometry imaging (DESI‐MSI) reveals auxin‐cytokinin signaling gradients and auxin response factor (ARF)/PLETHORA (PLT)‐mediated cell fate determination from quiescent center through meristematic, transition, and elongation zones. (F) Arabidopsis shoot and floral meristem development: WUSCHEL (WUS)‐CLAVATA3 (CLV3) feedback loop and ABC model. Spatial multi‐omics (transcriptomics + assay for transposase‐accessible chromatin using sequencing [ATAC‐seq]) reveals stem cell maintenance via WUS‐CLV3 negative feedback loop and organ identity determination through MADS‐box gene expression patterns (ABC model) defining sepal, petal, stamen, and carpel whorls. ARF, auxin response factor; Defb, β‐defensin; E, embryonic day; ECM, extracellular matrix.

Gametogenesis relies on precise crosstalk between germ cells and supporting cells. Conventional single‐cell sequencing fails to preserve the original spatial localization of cells in tissues, making it difficult to reveal the structural basis of the microenvironment. Spatial omics technologies enable in situ dissection of regulatory mechanisms underlying cell communication, signaling gradients, and fate determination in structures such as testicular seminiferous tubules and epididymal epithelium. This section first elaborates on their applications in the male reproductive system (testis and epididymis), uncovering the spatial regulatory mechanisms of spermatogenic cycle synchronization and sperm maturation (Figure 8A). Next, it discusses research advances in the female reproductive system (ovary), clarifying the microenvironment dependence of folliculogenesis and germ cell fate determination (Figure 8B). Finally, it analyzes the current technical challenges in this field.

In the testis, periodic synchronization of the seminiferous epithelium is a central process of male gametogenesis. Chakravorty et al. [323] performed Single‐cell‐level ST analysis on mouse testes using high‐resolution RNA seqFISH+, and constructed a three‐dimensional molecular atlas comprising 216,090 cells (Figure 8C). Within the seminiferous tubules, the authors identified a set of periodic transcriptional signatures that highly match the stages of the seminiferous epithelial cycle, enabling precise delineation of temporal progression during germ cell development. Further analysis revealed that the periodic transcriptional dynamics of Sertoli cells are synchronized with the developmental process of germ cells, and that such transcriptional rhythms can be stably maintained independently even in the absence of germ cells. Together, these findings uncover a previously unrecognized regulatory mechanism in spermatogenesis: Sertoli cells exhibit intrinsic periodic transcriptional fluctuations that temporally orchestrate and regulate the cyclic developmental process of germ cells. In the epididymal microenvironment, Zhang et al. [324] integrated single‐cell and spatial transcriptomics data to systematically characterize the region‐specific expression gradients of the β‐defensin (*Defb*) gene family in the caput, corpus, and cauda epididymis. Importantly, this study further confirmed by spatial localization that *Defb* mRNA can be delivered directionally from the epididymal epithelium to the sperm surface via epididymosomes, providing direct spatial evidence for regional molecular modifications during sperm maturation. Together, these two studies reveal the autonomous rhythmic mechanism underlying cell cycle synchronization in the testis and the directionality of regionalized substance transport in the epididymis, respectively.

Female germ cell development involves complex cell–cell interactions, and precise microenvironmental localization is essential from embryonic specialization to the establishment of adult follicular reserve. Below are the findings of spatial omics in ovarian development research of model organisms and mammals: A study in Drosophila that combined spatial transcriptomics and functional validation [325] showed that the glycoprotein Dally spatially restricts the enhancement of BMP/Dpp signaling to maintain the undifferentiated state of primordial germ cells, establishing a spatiotemporal coordination mechanism between germ cell fate determination and organ development. This highlights the structural role of the extracellular matrix in shaping signaling gradients in the microenvironment. In mammalian models, a study on the entire developmental process of mouse ovaries [326] used single‐cell resolution spatial transcriptomics to construct a continuous spatiotemporal atlas from embryonic to adult stages. This uncovered the spatial arrangement of various cells during follicular reserve establishment, revealing the heterogeneity of ovarian cortical cell composition, and providing a molecular basis for the spatial regulation of follicle dormancy and activation. Research on embryonic ovaries of the large animal model pig [327] clarified the cell migration rules during early oogenesis. Mainly, germ cells exhibit a gradient distribution dynamic from the cortex to the medulla, and this spatial rearrangement is coordinately regulated by the *NOTCH* signaling pathway and specific extracellular matrix proteins. This verifies the spatial expression pattern of key signaling molecules and inferring the spatially dependent regulatory logic of meiosis initiation and folliculogenesis, providing a reference for cross‐species research.

However, the full application of spatial omics in gametogenesis research still faces technical bottlenecks, with commonalities and tissue‐specific challenges in male and female reproductive systems. First, the mismatch between spatial resolution and tissue structure. Testicular seminiferous tubules have a higher density of cells (10−20 μm) than the resolution of mainstream commercial platforms (e.g., Visium, resolution of 55−100 μm). This leads to mixed cell RNA signals in capture spots [328] and difficulty in distinguishing cell types and specific gene expression. Second, key cells such as spermatogonial stem cells and various meiotic stages of oocytes are rare and have low RNA content. For these cells, traditional in situ capture methods have insufficient transcript coverage, resulting in increased gene detection dropout rates and affecting the accuracy of regulatory network reconstruction. To address these limitations, next‐generation high‐sensitivity technologies (such as Decoder‐seq) have improved mRNA capture efficiency by nearly an order of magnitude through innovative dendrimer barcoding design. This enhances the detection of low‐abundance transcripts while maintaining single‐cell resolution, providing new possibilities for rare cell localization and microenvironmental interaction analysis. However, their practical application effects in dense tissues such as the testis still need further verification.

#### Embryonic organogenesis

Mammalian embryonic development involves complex morphogenesis from gastrulation to organogenesis, governed by spatiotemporally precise molecular regulation. ST preserves the three‑dimensional embryonic structure while capturing dynamic gene expression, providing a critical tool for dissecting cell fate determination and tissue patterning.

During gastrulation, Cheng et al. [329] used Stereo‐seq to generate a continuous 3D spatiotemporal transcriptomic atlas of mouse embryos from E5.5 to E13.5 (Figure 8D). This study characterized mesoderm induction and the progressive refinement of spatial gene expression boundaries during body axis formation. It provided high‐resolution insights into the balance between developmental plasticity and pattern stability. During neurulation, Kumar et al. [288] applied Slide‐seq to profile whole embryos at E8.5−E9.5. They focused on anterior‐posterior axial differentiation of the neural tube. These two studies complement each other in platform and focus, jointly show the global molecular landscape of the gastrula‐to‐neurula transition. During organogenesis, Qu et al. [330] used the Visium platform to construct a 3D molecular architecture of major organ primordia in E13.5 mouse embryos, revealed the cell type composition and spatial heterogeneity in the heart, liver, lung, and other organs. They revealed that organ identity is determined not only by cell‐intrinsic programs but also by spatial constraints from local microenvironmental signals. Another study using single‐cell transcriptomics and computational modeling [331] focused on the early stage of lineage specification. It explored how the blastocyst inner cell mass differentiates into the epiblast and primitive endoderm, and revealed the regulatory mechanisms behind this process.

The aforementioned studies have shown that spatial omics can be very helpful in studying embryonic development. Furthermore, integrating multiple spatial omics data can improve the study of mammalian embryonic development. Some studies have constructed a molecular reference framework for mammalian embryonic development by integrating multi‐stage and multi‐scale spatiotemporal omics data. This framework supports *in vivo* mechanism research and provides key molecular benchmarks for constructing and validating in vitro embryo‐like models [332]. Importantly, subsequent technological upgrades in spatial omics have led to the introduction of Stereo‐seq technology. Stereo‐seq has nanometer‐level resolution and a wide field of view, solving the problem of low‐resolution based on sequencing idle time. It has also been used in practice to construct a complete spatiotemporal transcriptome map covering mouse organogenesis.

However, analysis using only mice is insufficient. Mice are the basic model for mammalian development, while humans and non‐human primates differ significantly in developmental timing and organogenesis patterns. A recent study [333] integrated Single‑cell and spatial transcriptomics to generate a panoramic cell type atlas of human embryonic development. This elucidated molecular programs of axial patterning and developmental transitions, quantitatively identified key differences between human and mouse development, and provided insights into congenital developmental defects. Another study [334] focused on human gastrulation and early neural development, revealing spatial organization of the neural tube, molecular programs underlying the transition from neuroepithelium to radial glia, and species‐specific regulatory networks. To better understand human development, ST has recently been used to study non‐human primate embryos to reveal primate‐specific developmental mechanisms. In marmoset embryos, in situ analysis [335] revealed the molecular framework underlying cell diversity and axial patterning during early gastrulation, identifying conserved and species‐specific regulators. In cynomolgus monkeys, high‑resolution ST [336] finely mapped the three germ layers during gastrulation, highlighting the central roles of spatially restricted gene expression and region‑specific signaling in lineage commitment and germ layer morphogenesis. Compared to mice, primate embryos exhibit greater cellular subtype diversity and more refined spatial segmentation during gastrulation.

### Applications of spatial omics in plants

Studies in animal development have demonstrated that spatial omics technologies can effectively dissect the spatial regulatory mechanisms underlying densely packed tissues and dynamic developmental processes. Similar principles apply to plant developmental biology. Plant organogenesis relies on the continuous activity of meristems. However, cell fate determination in root apical, shoot apical, and floral meristems is deeply embedded in the spatial heterogeneity of hormone gradients, signaling molecule distribution, and cell wall properties. However, the physical barrier of plant cell walls, the three‑dimensional complexity of tissue architecture, and the metabolic specificity conferred by photoautotrophy pose unique challenges for the application of spatial omics in plants. Below, we discuss the research progress of spatial omics technologies in plant root development, shoot apical meristem (SAM) maintenance, and floral organogenesis.

#### Root development

Plant root development requires precise coordination between cell division, differentiation, elongation, and pattern formation, and its regulatory mechanisms lie within the microenvironmental gradients of the root tip's three‐dimensional space. This section begins with the basic applications of ST, elaborates on its role in elucidating cell lineage trajectories and hormone distribution, and finally discusses its unique advantages in revealing plant‐microbe symbiotic relationships.

Arabidopsis thaliana has become the preferred model system for establishing technical pipelines and validating analytical strategies due to its simple root structure, clear cell lineage, and convenient genetic manipulation. Using ST, researchers have successfully constructed a continuous panorama of gene expression in the Arabidopsis root tip, spanning from the quiescent center, meristematic zone, transition zone, to elongation zone [337] (Figure 8E). This study not only accurately identified key transcription factors regulating stem cell maintenance, cell fate determination, and differentiation processes, but also revealed the gradient distribution of these regulatory factors along the longitudinal axis of the root tip [338]. The study found that these transcription factors exhibit a precise spatial pattern. Namely, a gradual decrease from the root tip to the root base, which directly determines the identity and differentiation fate of cells in different root zones.

In woody plant research, time‑series ST analysis of adventitious root regeneration in poplar (Populus) [339] revealed a complete developmental trajectory from callus to functional root. This study identified clusters of cytokinin‐responsive genes that are co‐activated at specific spatiotemporal stages, revealing a causal relationship between the spatial distribution of hormone signals and the initiation of organogenesis. This provides key insights into the molecular basis of vegetative reproduction in woody plants and demonstrates the potential of ST in agricultural biotechnology.

Furthermore, the combined application of spatial metabolomics (e.g., MALDI‑MSI or DESI‑MSI) and ST has allowed direct observation of the in situ distribution of hormones (e.g., auxin, cytokinin) and secondary metabolites in root tissues, and their correlation with gene expression in adjacent cells [340]. Such cross‑modal associations not only validate the spatial consistency between the expression of hormone biosynthetic genes and their product distribution, but also uncover precise matching between metabolite diffusion ranges and signaling response domains. This helps to reveal the complete regulatory cascade of the gene expression/metabolite distribution cellular response.

In the field of plant‐microbe interactions, one group [341] found that the spatial expression patterns of specific sugar transporters (*SWEETs*) in roots directly shape the colonization pattern of the rhizosphere microbial community. This study highlights the key role of spatial metabolite distribution as a “niche constructor.” Mainly, that plants actively and selectively recruit beneficial microbes by precisely controlling the spatial localization of sugar secretion, thereby establishing a mutualistic spatial structure. This discovery extends the application of spatial multi‑omics from developmental biology to ecological interaction research, demonstrating its potential in dissecting interspecies spatial relationships.

Spatial multi‑omics exhibits unique advantages for dissecting the intimate symbiotic relationships between roots and microbes, particularly when studying specialized interaction interfaces involving only a small number of cell types. Arbuscular mycorrhizal (AM) symbiosis is a key pathway for plants to acquire nutrients such as phosphorus, but its core interface—the arbuscule—resides exclusively in a small subset of root cortical cells. This highly restricted cellular interaction makes it difficult for traditional bulk sequencing to capture the molecular details, whereas ST can precisely identify these rare interacting cells in situ and resolve their specific molecular responses. By combining single‑nucleus RNA sequencing (snRNA‑seq) and spatial RNA sequencing, researchers have successfully generated a co‑transcriptomic atlas of both plant and fungal partners during AM symbiosis [342]. This study clearly demonstrates spatiotemporal coordination of gene expression at the symbiotic interface: Here, the early infection, arbuscule development, and functional maturation stages correspond to distinct transcriptional programs in the host plant, which are strictly spatially confined within cortical cells. A suite of symbiosis‑related genes specifically expressed at particular infection stages and spatial positions was identified, providing spatial evidence to understand the molecular dialogue of mutualism.

Similarly, spatiotemporal transcriptomic analysis of nodule organogenesis in Lotus japonicus [343] established a molecular event map covering the entire process from rhizobial infection, nodule organogenesis, to the maturation of nitrogen‑fixing function. This study not only identified spatial molecular markers for each stage of nodule development, but also uncovered key regulatory nodes during the establishment of symbiotic nitrogen fixation. This provides valuable gene resources and regulatory network information for transferring nitrogen‑fixing capacity to non‑leguminous crops with important agricultural application prospects.

Nevertheless, the application of spatial multi‑omics to plant root research still faces technical challenges unique to plant tissues, arising from the physical barrier of cell walls and the three‑dimensional structural complexity of root apices. The presence of plant cell walls makes tissue permeabilization, sectioning, and molecular capture more difficult than in animal tissues. The dense structure of cell walls impedes the penetration of probes and capture reagents, often resulting in limited spatial resolution, reduced RNA capture efficiency, and loss of molecular signals, especially in highly lignified mature root tissues. The effective resolution of current commercial platforms (e.g., Visium) is typically around 55 μm, much larger than the diameter of individual plant cells (approximately 10–20 μm). This resolution mismatch means that a single capture spot often contains multiple cells, generating “mixed signals” that require sophisticated computational deconvolution algorithms (such as SPOTlight, RCTD, cell2location) to resolve cell‑type composition [344]. However, the application of these algorithms in plant tissues is limited by the scarcity of reference datasets. Also, the uncertainty of plant cell‑type marker genes and their resolution accuracy remain to be improved.

To address these challenges, researchers are developing plant‐specific methodologies. Enzymatic digestion with cellulase‐pectinase cocktails effectively degrades rigid plant cell walls, yielding protoplasts. Subsequent single‐cell RNA sequencing can be integrated with high‐resolution spatial barcoding or computational spatial reconstruction approaches [345]. Although single‐cell profiling inherently sacrifices original spatial information, computational integration with established tissue architectures or reference atlases enables the inference of cellular spatial coordinates within tissues [346].

PHYTOMap [347] is a multiplex FISH‐based technology that uses probe sets targeting endogenous mRNAs to perform in situ expression analysis of hundreds of genes in whole‐plant tissues at single‐cell resolution without relying on transgenic markers. This method overcomes the low‐throughput limitation of traditional FISH and provides a high‐resolution, sequencing‐independent alternative for plant spatial transcriptomics. However, the optical opacity of intact plant tissues restricts imaging depth, limiting access to deep cortical layers in organs such as root tips, SAMs, and nodules. To circumvent this constraint, tissue clearing protocols such as ClearSee [348] and M2WISH [349] employ optimized cell wall digestion enzyme cocktails to achieve optical transparency while preserving RNA integrity and three‐dimensional architecture. Integration of these clearing methods with multiplex FISH (e.g., PHYTOMap) or hybridization chain reaction (HCR) enables single‐cell resolution gene expression profiling in intact tissues where traditional mechanical sectioning fails to capture continuous spatial information [350]. This combined approach is particularly critical for studying arbuscular mycorrhizal symbiosis and nodule organogenesis, where the interaction interfaces reside in deep tissues inaccessible to standard sectioning techniques.

#### Shoot and floral development

The SAM serves as the origin for the development of all above‐ground plant organs. Stem cell maintenance and organogenesis within the SAM rely on the spatially dynamic homeostasis of key regulatory circuits, including the *WUS‐CLV3* feedback loop (Figure 8F). Flower development introduces an additional layer of complexity through organ identity specification, which requires precise spatial boundary control of the combinatorial expression patterns of MADS‐box genes across the four floral organ whorls (sepals, petals, stamens, and carpels). This section discusses the applications of spatial omics in SAM stem cell maintenance and floral organ identity determination, elaborates on the multi‐layered regulatory logic spanning transcriptomics, epigenomics, and metabolomics, and explores the potential of spatial proteomics in deciphering genotype‐phenotype mapping (Figure 8F).

In studies of Arabidopsis flower development, researchers employed single‐nucleus RNA sequencing (snRNA‐seq) combined with spatial reconstruction algorithms to construct a 3D gene expression atlas of the floral meristem [351]. Studies have revealed significant transcriptomic differences between stem cells in the central region of floral meristems and their differentiated daughter cells in the peripheral region. This is particularly critical for understanding the spatially dynamic homeostasis of key stem cell regulatory mechanisms, such as the WUSCHEL (*WUS*)‐CLAVATA3 (*CLV3*) feedback loop. In this circuit, WUS protein migrates from the organizing center to the central zone to activate *CLV3* expression, while the *CLV3* peptide signal in turn represses *WUS* expression, forming a precise negative feedback loop. ST data have revealed the spatially separated yet overlapping domains of WUS and *CLV3* expression, providing molecular‐geographic evidence for the homeostatic maintenance of stem cell number.

In floral organ development, ST enables the dissection of the precise spatiotemporal sequence of floral organ primordium formation and the expression boundaries of key regulatory genes—especially those of the MADS‐box gene family. Flower development involves the precise arrangement of four whorls of organs: sepals, petals, stamens, and carpels. The establishment of this spatial pattern depends on the accurate interpretation of positional information and the regionalized specification of cell fate. The classic ABC model established the molecular basis for this patterning: the combinatorial expression of three classes of MADS‐box genes (A, B, and C) in the four whorls determines floral organ identity (A alone: sepals; A + B: petals; B + C: stamens; C alone: carpels). However, traditional models were largely based on genetic mutations and in situ hybridization data, lacking a systematic understanding of the precision of gene expression boundaries and cellular heterogeneity. ST allows for the precise mapping of these genes' expression domains across different floral developmental stages and reveals the dynamic changes of their expression boundaries. For example, during Phalaenopsis orchid floral organogenesis, a spatiotemporal atlas generated using the Visium platform [352] uncovered the complexity of MADS‐box gene expression patterns. In short, the sharp boundaries predicted by the conventional ABC model instead exhibit gradient changes and cellular heterogeneity during actual development, with overlapping and dynamically adjusted expression domains for several genes. This suggests that floral organ identity specification may be more flexible than the classic model implies.

Furthermore, integrating spatial transcriptomics with chromatin accessibility data (e.g., spatial ATAC‐seq) elucidates how the epigenetic microenvironment regulates the execution of the ABCDE model genes. MADS‐box proteins execute the genetic program described by the ABC model by forming multimeric tetrameric complexes known as floral quartets. ST analysis provides cell‐resolved spatial expression evidence for the functional validation of these protein complexes, allowing researchers to test the correspondence between specific tetramer combinations and cell fate. Precise spatial dissection of these gene expression patterns helps reveal the specific mechanisms by which MADS‐box genes act in the formation of different floral organs, particularly how they determine organ identity through fine‐tuned quantitative regulation rather than simple binary on/off states.

Spatial metabolomics and proteomics have also provided new insights into the development of plant stems and flowers. Plant glandular trichomes are specialized structures for the synthesis and storage of secondary metabolites, and their development is closely related to the dynamic biosynthesis of metabolic compounds. The spatial distribution and density of these structures directly determine the chemical phenotype of plants. However, traditional methods have struggled to elucidate the in situ connection between their development and metabolism. Spatial metabolomics can directly observe the dynamic in situ accumulation of metabolites during glandular trichome development and correlate it with the gene expression status of neighboring cells [353]. This approach reveals that metabolite accumulation is not homogeneous but is instead stage‐specific and spatially restricted, providing a new strategy for dissecting the molecular mechanisms governing trichome development. By integrating spatial transcriptomics and metabolomics data, researchers can establish the complete cascade of “gene expression‐enzyme activity‐metabolite accumulation”, leading to a more comprehensive understanding of the spatiotemporal distribution and regulatory logic of plant secondary metabolites [354, 355]. For example, the expression peaks of specific terpene synthase genes correspond precisely to the accumulation of their cognate metabolites in glandular head cells, validating the tissue specificity of the biosynthetic pathway. In Perilla frutescens, quantitative spatial phenotypic analysis of peltate trichome developmental patterns, combined with metabolomic data, revealed spatial correlations between trichome density, developmental stage, and the production of metabolites such as rosmarinic acid, providing targets for the directed improvement of chemical phenotypes in medicinal plants.

On the other hand, advances in spatial proteomics have made it possible to directly visualize the in situ distribution of proteins. Although plant spatial proteomics research is still in its early stages [356], its potential is enormous. Indeed, proteins are the direct executors of gene function, and their spatial distribution, modification status, and interaction networks ultimately determine cellular behavior. In the future, the deep integration of spatial proteomics with transcriptomics and metabolomics will provide authoritative tools for elucidating the complete cascade of genotype‐phenotype mapping during stem and flower development.

### Future challenges and conclusions

Spatial omics applications in animal and plant developmental biology have revealed spatial regulatory mechanisms inaccessible to conventional methods [357, 358]. However, technical challenges persist due to tissue specificity: limited resolution in dense animal tissues, inefficient capture of rare germ cells, and permeabilization difficulties from plant cell walls restrict analytical depth in specific scenarios. Future research should address these bottlenecks—for example, integrating ultra‐high‐resolution techniques for rare cell localization or developing plant‐specific methods for single‐cell‐resolution analysis. As technical boundaries expand, spatial omics will advance from descriptive profiling to functional dissection, strongly supporting theoretical breakthroughs in developmental mechanisms and practical applications in agricultural biotechnology.

## FOUNDATION MODELS FOR SPATIAL OMICS

**Figure 9:**
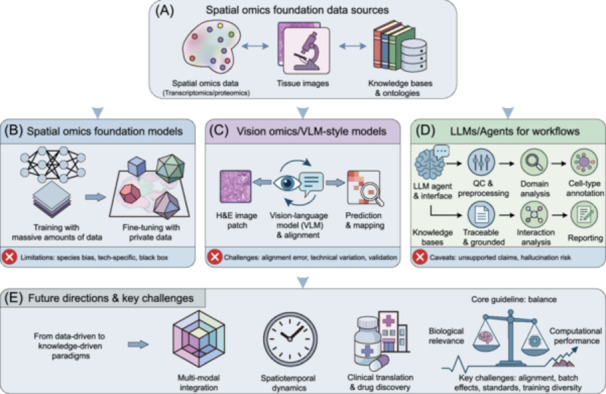
Large‐scale models in the field of spatial omics. (A) Data sources for training such models: spatial omics data, tissue images, as well as knowledge bases including literature and bioinformatics software. (B) Spatial omics foundational model. (C) Vision‐omics model. (D) LLM/Agent. (E) Challenges and future directions of large‐scale models in spatial omics. LLM, Large Language Model.

This section examines the current applications and challenges of large‐scale pre‐trained models in spatial omics research including foundation models [359], visual language systems, and large language models—in spatial omics research. Spatial omics data for model development are primarily derived from data matrices, tissue images, relevant literature, and knowledge bases (Figure 9A). Based on their architectural characteristics, these models can be broadly classified into three categories: spatial foundation models, visual omics models, and large language models. Spatial foundation models learn reusable representations of spatial molecular data through multi‐sample training. Visual omics models establish mappings between histological images and molecular profiles. Large language models integrate external tools and knowledge bases to coordinate analytical workflows, interpret results, and generate reports. Despite differences among these model types, they all depend on specific assumptions such as accurate data pairing, authentic knowledge sources, and appropriate preprocessing and modeling methods. Moreover, the applicability of a model to real experimental scenarios—such as sequencing‐ and imaging‐based transcriptomics [360], multiplex spatial proteomics [318, 319], and community standards [361], is a core criterion for researchers when selecting methods. Currently identified application constraints include limited labeled data, diverse data types that are difficult to reuse, challenges in cross‐modal pairing, lack of streamlined processing, and excessive computational resource consumption in some models [107, 115, 116, 128, 129, 362]. The following sections elaborate on these models, beginning with their classification.

### Spatial omics foundation models

Spatial omics foundation models (Figure 9B) rely on large‐scale sample data to learn organizational spatial structural features. The term “foundation” does not denote a fixed model architecture [363, 364], but rather the model's ability to be universally reused across organizations and platforms. The model's practical performance is primarily influenced by the quality of training data, architectural design differences, and experimental technical variations. Several limitations remain for this model type: first, existing training data are heavily concentrated on human and mouse samples, limiting adaptability to niche research fields and underscoring the urgent need for universal models capable of cross‐species transfer learning. Second, most model architectures are customized for a single sequencing technology, hindering efficient compatibility and reuse of resolution data produced by different experimental techniques. For example, some models analyze spatial regions using single‐cell resolution combined with neighborhood information, while others use local regions as units to identify tissue microenvironments and cell ecological niches [365, 366, 367]. Third, many models overlook inherent experimental issues such as sequencing noise, cell segmentation errors, and tissue deformation distortions during architecture design, often requiring specialized processing tools to mitigate data artifacts. However, standardized guidelines for combining these tools are lacking, and the black‐box nature of models remains prominent, further limiting the stability and generalizability of analyses.

Different recent systems emphasize different design goals: building neighborhood representations that adapt to new data with minimal retraining [368, 369], aligning tissue images and molecular measurements during training [370], reducing computational cost [371], modeling spatial proximity and cell interactions [372], learning correspondences between different data modalities [373], or using transformer‐based architectures with spatial structure built in [374]. This section focuses on two widely recognized models: Novae [369] and scGPT‐spatial [375].

Novae is a graph‐based ST foundational model, trained on nearly 30 million cells from spatial transcriptomics datasets covering human, mouse, and multiple tissue types including the brain. Its core advantage lies in zero‐shot [376] transfer capability and multi‐scenario adaptability. Researchers can complete spatial domain predictions without retraining from scratch. It supports batch effect correction across multiple slices to ensure consistency in cross‐slice analysis results. It is compatible with any gene combination, tissue type, and spatial omics technology, while covering key tasks such as spatial differential gene analysis, pathway analysis, and trajectory inference. Moreover, researchers can incorporate histological images and protein data for joint analysis. Tests show that fine‐tuning this model on the Mosta dataset takes about 8 h on a single NVIDIA GeForce RTX 4090, with the 32.0M‐parameter model delivering efficient performance in subsequent analyses, as reported in the original development study.

scGPT‐spatial is a ST foundation model based on the scGPT [377] Transformer architecture, with its core design—including a mixture‐of‐experts (MoE) decoder [378], a spatially aware sampling strategy, and continual pretraining techniques, trained on the large‐scale SpatialHuman30M dataset. The SpatialHuman30M dataset is a comprehensive ST resource containing 30 million ST profiles, generated using both imaging‐based and sequencing‐based technologies including Visium, Visium HD, Xenium, and MERFISH, with tissue samples derived from normal state, cancerous state, and other diseased states. Its main advantage lies in efficiently unifying multi‐platform data and excelling in downstream tasks such as multimodal integration, single‐slice clustering, cell type deconvolution, and gene expression imputation. It also supports zero‐shot inference, offering strong generalizability and controllable computational cost. The model's key contributions include the construction of the first large‐scale ST dataset and the proposal of a modeling scheme that adapts to protocol heterogeneity and spatial relationships, reducing the reliance on task‐specific fine‐tuning. From the perspective of personalized applications, the platform provides a general model pretrained on 33 million normal human cells, as well as various specialized pretrained models for brain, blood, heart, lung, kidney, and pan‐cancer research, enabling researchers to flexibly select according to their own research needs.

In practice, ensuring biological significance at each process step and minimizing technical defects and algorithm errors remain critical priorities, as emphasized in interdisciplinary consensus. Most software developers focus on information mining in expression matrices, neglecting key biological information such as signaling pathways, partial database descriptions, and species‐specific characteristics. However, multi‐omics data inevitably brings interpretation difficulties, making it a promising direction for computational biology developers to prioritize interpretability over mere model architecture innovation, with potential to address current limitations in biological relevance.

### Vision‐omics/VLM‐style models linking histology with gene expression

Vision‐omics models link tissue images (typically H&E‐stained sections) to spatial gene expression data (Figure 9C). These models support key analytical goals: predicting gene expression in unmeasured regions, finding and mapping similar structures between images and gene measurements, prioritizing regions for detailed study, and aligning multiple tissue sections. Two main challenges affect performance. First, correspondence error: tissue images and expression measurements may not align perfectly due to tissue distortion and measurement limitations. Second, technical variation: staining protocols, imaging equipment, and data‐collection locations introduce systematic differences that models must handle. Therefore, future model evaluation should not rely merely on held‐out test data from a single study, but should further examine its generalization ability across different batches and experimental conditions.

Technically, images and expression data have different structures. Specifically, gene expression is measured at individual spots or cells, while images consist of multi‐scale visual patches. Most models process tissue images using image‐analysis methods (trained on pathology images), convert the resulting features to match expression resolution, add spatial context from neighboring cells, and use alignment approaches that acknowledge uncertainty in the pairing between images and expression. Published systems use several different design choices. Some methods train on paired image and expression data to learn alignments between them. Others combine strong image‐analysis methods (from pathology) with expression‐specific modules. Generative models (using diffusion‐based approaches) [379] can represent uncertainty in expression prediction [380], and interactive tools allow researchers to query and explore predictions.

OmiCLIP [381], as a representative of visual‐omics models (based on ST‐bank dataset), uses the ViT visual encoder and Transformer transcriptome encoder to extract features from H&E pathology images and gene expression, respectively. It is trained with cross‐modal contrastive learning through CoCa technology as the core, building a cross‐modal representation model based on millions of spatial transcriptome‐image sample pairs. Visual‐text pathology large model CONCH [382] also adopts a similar CoCa‐based multimodal training scheme. The LOKI platform, relying on the embeddings from OmiCLIP, has developed five major functional modules: Loki‐Align (cross‐modal alignment), Loki‐Annotate (automatic annotation), Loki‐Decompose (cell type decomposition), Loki‐Retrieve (reverse retrieval), and Loki‐PredEx (gene expression prediction). This achieves a breakthrough in directly interpreting molecular information from pathology images—both addressing the limitation of traditional pathology of “seeing morphology but not molecules,” significantly reducing the cost of ST experiments, and uncovering the molecular diagnostic value of pathology slides. It also features strong generalization and high scalability, providing a general foundational framework for AI‐pathology integration, spatial omics research, and precision diagnosis.

In view of the characteristics of such models, future evaluation systems should include performance tests across different patients and data collection centers, and quantitatively measure model sensitivity to variations in staining protocols and changes in imaging equipment [383, 384]. Meanwhile, stress tests with deliberate misalignment between images and gene expression should be designed to distinguish two types of errors: alignment positional errors (where the biological signal is genuine but spatially misplaced) and biologically incorrect prediction results. The same approach can be extended to spatial proteomics, where proteins are detected in tissue images. Recent systems like HEX and HistoPlexer show that combining tissue images with predictions of protein and gene expression can improve clinical decision‐making [385]. However, these systems face identical alignment and technical‐variation challenges as gene‐expression models and require the same careful evaluation and reporting.

### LLMs/Agents directly for spatial omics workflows

Spatial biology research requires handling complex high‐dimensional data and relies on distributed computing methods that require substantial manual intervention. These methods are not only inefficient but also difficult to adapt to diverse datasets and biological contexts. Even foundation models and vision models cannot be quickly used by research novices. Therefore, LLM/agents for spatial omics (Figure 9D) have emerged. These models deliver impressive performance. Nevertheless, researchers should be aware that such agents still face substantial obstacles in real‐world vertical‐domain applications, particularly the stringent requirements for data quality and computational resources. Consequently, current spatial omics agents cannot yet match the user experience and maturity of state‐of‐the‐art general models used in daily scenarios. Even so, this field remains a major developmental direction for the future. This section is mainly discussed based on publicly available spatial omics agents, such as SpatialAgent [386] and CellAgent [387].

SpatialAgent is an artificial intelligence agent specifically designed for spatial omics research, compatible with Claude, GPT, and Gemini large‐scale language models by configuring corresponding API keys. It adopts a three‐tier core architecture: a memory module that stores long‐term goals and short‐term execution steps, a planning module that decomposes complex tasks through chain reasoning, and an action module that executes tool calls and database interactions. At present, the developers have fully validated its core capabilities: in terms of gene panel design, through reference dataset matching and cross‐database verification, it outperforms traditional algorithms and 90% of human experts, demonstrating stronger human artificial intelligence collaboration capabilities. In cell annotation, through multimodal reasoning, its consistency with the gold standard reached 82.3%, surpassing existing tools in terms of efficiency and cost‐effectiveness. In addition, it can generate scientific hypotheses and detailed analysis reports, discover key mechanisms previously overlooked in mouse colitis models, and enhance data analysis and clustering performance by optimizing gene panels in wet experiments of prostate cancer mouse models. We propose three practical usage guidelines. First, to reduce resource consumption, users may limit each code execution to ten lines or fewer. Secondly, to ensure reliable performance, task descriptions should clearly specify species, tissue types, analysis objectives, and data sources to avoid ambiguity. Finally, handling complex multi‐step workflows should be divided into continuous subtasks to minimize LLM inference errors. Through these three strategies, users can reduce resource consumption and large model error rates.

CellAgent is built on a multi‐agent collaborative decision‐making framework mimicking human experts' analytical thinking, comprising three LLM‐driven core agents: Planner, Executor, and Evaluator, handling task decomposition, code execution, and result optimization respectively. It integrates the sc‐Omni toolkit with Squidpy, Harmony, CellTypist, and other tools to support batch correction, cell annotation, trajectory inference, and spatial transcriptomics analysis. Evaluated on over 60 datasets across eight tasks, CellAgent completes analysis in 8 min on average, faster than human experts (13 min), with comparable quality and a 96% code execution success rate, far exceeding GPT‐4's 23.875%. It achieves state‐of‐the‐art performance in key tasks: top‐ranked batch correction, 85% cell annotation accuracy matching experts, best trajectory inference, highest spatial domain recognition on DLPFC data, and 17% higher spatial imputation accuracy than the second‐best method. Its success relies on domain and error‐case datasets for spatial omics knowledge understanding, and reward mechanisms for multi‐role collaboration, with quantitative metrics guiding self‐optimization of the three agents.

As multifunctional and scalable large language model agents in the field of bioinformatics, both of these agents can achieve automation of analysis in the field of spatial omics. These developments suggest promising opportunities for automating spatial omics analysis. However, we need to more clearly understand how to address the issue of hallucinations in large models and how to verify that the analyses or conclusions provided by the agents are reasonable and real. Currently, there are three main targeted solutions. First, although automation is feasible, it is recommended that researchers verify results step by step to prevent going further down the wrong path, wasting computational resources and time. Secondly, by using a multi‐agent voting strategy, the most reasonable and correct analysis results can be obtained. Finally, human‐computer interactive discussions and debates may be more reasonable.

### Limitations, challenges, and future directions

In summary, spatial omics models are evolving from data‐driven to knowledge‐driven paradigms (Figure 9E). Current research hotspots in this field include: unified modeling of multi‐modal data, improved accuracy of cell analysis and efficiency of drug target discovery through the integration of biological knowledge, and accelerated bioinformatics analysis for researchers enabled by large language models. Nevertheless, such models still face multiple technical challenges: alignment error, batch effects, data standards, training diversity, and other common issues. Currently, computational issues such as registration errors and batch effects are continuously being optimized, and large‐scale pre‐trained models with increasingly powerful capabilities have emerged, leading to gradual improvements in these computational and statistical problems. However, more critical and intractable challenges remain: the black‐box effect, significant difficulties in integrating biological knowledge into data‐driven algorithms (specifically, balancing computationally optimal solutions with biological plausibility), the construction of pre‐training datasets, and adapting world‐class models to spatial omics research. These issues are substantial and urgently require collaborative efforts from researchers across multiple disciplines.

Future spatial omics models will take AI foundation models as the core engine, deeply integrate multi‐omics data, enable spatiotemporal omics research across time and space, achieve the leap from static profiling to spatiotemporal dynamic inference, and accelerate translation toward clinical standardization and drug discovery [388, 389, 390, 391]. For instance, TIMES [392], a Chinese liver cancer prediction system developed based on multi‐omics and patient data, has realized the prediction of recurrence risk in hepatocellular carcinoma. It can be seen that large models based on spatial omics are playing an increasingly important role in the life sciences industry.

## SUMMARY AND PROSPECT

Against the backdrop of rapid advances in artificial intelligence, spatial omics technologies enable the precise characterization of molecular spatial distribution patterns and cell–cell interactions. These innovations have propelled biological research beyond the traditional scope of component identification and into a new dimension focused on spatial‐functional integration. This review systematically reviews research progress in spatial omics from multiple perspectives, comprehensively covering core experimental techniques, key computational tasks, supporting software tools, cutting‐edge developmental trends, and emerging algorithmic models (Table 5). It also summarizes the applications of such technologies in diverse scenarios, including animal development, plant growth regulation, neural circuit mapping, disease pathological research, and clinical diagnosis and therapy, while envisioning the broad prospects of large‐scale AI‐driven pre‐trained models in this field. This review aims to provide experimental researchers in spatial omics with practical technical workflows, analytical software, and automated analysis models. For algorithm developers, it organizes tool collections, evaluation frameworks, and guidance for the development of cutting‐edge methods in various subfields.

**Table 5:** Software recommendations for cutting‐edge research directions.

Task	Software	Language	Advantages
3D reconstruction	Spateo [194]	Python	Embryo 3D molecular hologram, reveals multi‐scale spatial biology, links morphogenesis and molecular dynamics, supports spatiotemporal quantification
	SpatialZ [195]	Python	Supports virtual slicing/3D rendering/spatial query, cross‐platform compatible, extensible to multi‐omics
Temoral alignment	moscot [218]	Python	Aligns multi‐time‐point spatial omics, supports > 500k cell datasets, incorporates growth/apoptosis gene prior knowledge
	DeST‐OT [219]	Python	Validated on axolotl data, models growth/apoptosis with triplet loss (no growth/apoptosis gene prior knowledge)
Dynamics reconstruction	OSDR [220]	Python	Estimates division/clearance rates, maps neighborhood to state space, constructs phase portraits to identify stable/unstable points
	StVCR [223]	Python	Addresses cross‐time‐point alignment difficulty/cell population imbalance, end‐to‐end reconstructs differentiation/migration/proliferation/apoptosis dynamics
Foundation models	Novae [369]	Python	Removes batch effects, identifies spatial domains, supports multi‐downstream analyses and multi‐modal integration
	ScGPT‐spatial [375]	Python	Adapts to multi‐downstream tasks: cell annotation, multi‐batch/multi‐omics integration, perturbation prediction, gene network inference, etc.
Vision‐omics model	OmiCLIP [381]	Python	Tissue registration/region annotation/cell deconvolution, retrieves transcriptomic data and predicts spatial expression profiles using only H&E sections
Expert agent	SpatialAgent [386]	Python	An autonomous AI agent for spatial biology
	CellAgent [387]	Python	LLM‐driven multi‐agent framework for automated scRNA‐seq data analysis

Spatial omics research should be centered on scientific questions and clinical needs, with researchers from various fields advancing collaboratively based on their respective roles. Biologists should focus on specific scientific questions and rationally select appropriate technical platforms: for studies on tissue macrostructure and regional heterogeneity, low‐resolution, large‐field‐of‐view ST technologies are recommended; for investigations into the cellular microenvironment and cellular neighborhood interactions, subcellular‐resolution or near‐Single‐cell‐resolution techniques are more suitable. During research, biologists should integrate experimental and computational approaches and adopt data‐driven strategies to reduce resource waste caused by repeated experiments. Meanwhile, as all algorithms have inherent limitations, researchers should improve analytical reliability through multi‐software comparisons and visual validation. Strengthening big data visualization competence is also a key skill for experimental biologists, who should therefore proactively engage in in‐depth cooperation with computational scientists. Computational scientists and bioinformatics researchers should shift their research focus from a data‐centric paradigm to one centered on spatial biology and clinical problems, striking a balance among methodological innovation, algorithmic robustness, and interpretability. They should prioritize the development of standardized and reproducible spatial analysis pipelines and unified data formats, promote the application of explainable artificial intelligence, and adopt benchmark evaluation methods tailored to real research scenarios. They should also systematically test the robustness of methods under realistic experimental conditions, including small sample sizes, low‐quality data, heterogeneous tissues, and formalin‐fixed paraffin‐embedded samples. Clinicians should design research protocols based on practical clinical problems, rather than merely conducting simple atlas‐based observations. They should actively explore spatial radiomics, promote the deep integration of spatial omics features with clinical imaging data such as hematoxylin‐eosin staining, magnetic resonance imaging, and computed tomography, and further develop non‐invasive or minimally invasive clinical predictive models.

Overall, the spatial omics field needs to establish interdisciplinary, cross‐platform unified standards and quality control systems covering the entire workflow—from sample processing, experimental execution, and data generation to analysis and result reporting—to improve the comparability and reproducibility of research findings. Technology developers should balance innovation with clinical translation. While pursuing higher resolution, additional omics modalities, and greater throughput, they must also consider cost control, detection speed, clinical accessibility, and regulatory compliance to ensure technological research aligns with practical application needs.

## AUTHOR CONTRIBUTIONS

**Haoxiu Wang**: Conceptualization, writing—original draft, validation, visualization, writing—review and editing, project administration. **Xinwang Yang**: Writing—original draft, validation, visualization, writing—review and editing. **Siheng Wang**: Writing—original draft, validation, visualization, writing—review and editing. **Zhe Yang**: Writing—original draft, visualization, writing—review and editing. **Xiuhui Yang**: Writing—original draft, visualization. **Yutong Yang**: Writing—original draft, visualization. **Zirong Li**: Writing—original draft, visualization. **Yuqi Ren**: Visualization. **Qianqian Zhang**: Visualization. **Bowen Zhao**: Writing—original draft. **Jingming Xiao**: Visualization. **Yidong Wang**: Writing—original draft. **Junhao Dong**: Writing—original draft. **Zhenhao Kou**: Writing—original draft. **Jie Li**: Writing—original draft. **Liqun Yang**: Supervision. **Erhu Zhao**: Supervision. **Gregory Fonseca**: Writing—review and editing. **Ruibang Luo**: Writing—review and editing. **Mingyu Yang**: Writing—review and editing, writing—original draft. **Hongjuan Cui**: Writing—review and editing, writing—original draft. **Gengjie Jia**: Writing—review and editing, writing—original draft. **Dan Wang**: Writing—review and editing, validation. **Haoyang Li**: Writing—original draft, writing—review and editing, supervision. **Jun Ding**: Writing—original draft, writing—review and editing, supervision. **Zhiyuan Yuan**: Writing—original draft, writing—review and editing, project administration. **Haojing Shao**: Funding acquisition, visualization, writing—review and editing, project administration, supervision, conceptualization.

## CONFLICT OF INTEREST STATEMENT

The authors declare no conflicts of interest.

## ETHICS STATEMENT

No animals or humans were involved in this study.

## Data Availability

Supplementary materials (figures, tables, graphical abstract, slides, videos, Chinese translated version and update materials) may be found in the online DOI or iMeta Science (http://www.imeta.science).
